# Different integration site structures between L1 protein-mediated retrotransposition in *cis *and retrotransposition in *trans*

**DOI:** 10.1186/1759-8753-1-17

**Published:** 2010-07-08

**Authors:** Kenji K Kojima

**Affiliations:** 1 Department of Biological Sciences, Graduate School of Bioscience and Biotechnology, Tokyo Institute of Technology, 4259-B-21 Nagatsuta-Cho, Midori-Ku, Yokohama, Kanagawa 226-8501, Japan; 2 Genetic Information Research Institute, 1925 Landings Drive, Mountain View, CA 94043, USA

## Abstract

**Background:**

Long interspersed nuclear element-1 (LINE-1 or L1) is a dominant repetitive sequence in the human genome. Besides mediating its own retrotransposition, L1 can mobilize *Alu *and messenger RNA (mRNA) in *trans*, and probably also SVA and non-coding RNA. The structures of L1 copies and *trans*-mobilized retrocopies are variable and can be classified into three categories: full-length; 5'-truncated; and 5'-inverted insertions. These structures may be generated by different 5' integration mechanisms.

**Results:**

In this study, a method to correctly characterize insertions with short target site duplications (TSDs) is developed and extranucleotides, TSDs and microhomologies (MHs) at junctions were analysed for the three types of insertions. Only 5'-truncated L1 insertions were found to be associated with short TSDs. Both full-length and 5'-truncated retrotransposed sequences in *trans*, including *Alu*, SVA and mRNA retrocopies and also full-length and 5'-inverted L1, were not associated with short TSDs, indicating the difference of 5' attachment between retrotransposition in *cis *and retrotransposition in *trans*. Target sequence analysis suggested that short TSDs were generated in an L1 endonuclease-dependent manner. The MHs were longer for 5'-inverted L1 than for 5'-truncated L1, indicating less dependence on annealing in 5'-truncated L1 insertions.

**Conclusions:**

The results suggest that insertions flanked by short TSDs occur more often coupled with the insertion of 5'-truncated L1 than with those of other types of insertions *in vivo*. The method used in this study can be used to characterize elements without any apparent boundary structures.

## Background

Long interspersed nuclear element-1 (LINE-1 or L1) is the only active non-long terminal repeat (non-LTR) retrotransposon family in humans and constitutes one sixth of the human genome [1,2]. It encodes two proteins, open reading frame one protein (ORF1p) and open reading frame two protein (ORF2p). Both these proteins are essential for L1 retrotransposition [3]. ORF2p contains two enzymatic domains: endonuclease (EN) [4] and reverse transcriptase (RT) [5]. Retrotransposition-competent L1 copies are ~6000 bp in length [6,7], but most of the L1 copies on the human genome are severely 5'-truncated [1,8]. In addition, ~10% of the human L1 copies are rearranged to be composed of two segments: 5' inverted and 3' noninverted segments [9,10]. L1 copies are classifiable into three categories: full-length; 5'-truncated; and 5'-inverted insertions.

**Figure 1:**
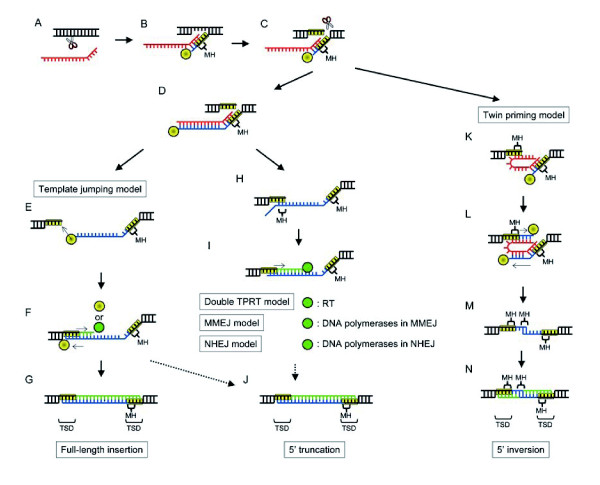
**Models for retrotransposition**. Five models have been proposed for three types of retrotransposition and all are variations of target-primed reverse transcription (TPRT); the first two steps are common in all models. (A) bottom strand cleavage; (B) target-primed reverse transcription; (C) top strand cleavage; (D) completion of first strand reverse transcription; (E) template jumping; (F) second strand cDNA synthesis by either RT or cellular DNA polymerase; (G) rejoining junctions; (H) annealing between top strand and first strand cDNA; (I) target-primed second strand cDNA synthesis with the usage of short sequence complementarity by either RT (double TPRT model), DNA polymerases in microhomolgy-mediated end-joining (MMEJ) pathway (MMEJ model), or DNA polymerases in non-homlogous end-joining (NHEJ) pathway (NHEJ model); (J) rejoining junctions; (K) annealing between top strand and template RNA; (L) target-primed reverse transcription from the top strand cleavage site; (M) RNA degradation and annealing between two cDNA strands; (N) DNA synthesis and rejoining junctions by unknown mechanisms.

The insertion of L1 is initiated by single-strand DNA cleavage catalyzed by EN [11,12] (Figure 1). L1 EN cleaves target DNA preferentially between TTTT and AA on the bottom (complementary) strand [4,13]. The cleaved 3'-end is used as a primer to initiate reverse transcription catalyzed by RT: this process is called target-primed reverse transcription (TPRT). The other DNA strand could be cleaved by EN during or after first-strand cDNA synthesis, followed by the second-strand cDNA synthesis. The second strand is generally cleaved downstream from the cleavage site of the first DNA strand. This distance causes target site duplications (TSDs), short direct repeats flanking both sides of L1. All variations of L1 retrotransposition (full-length, 5'-truncated and 5'-inverted insertions) are considered to be achieved by TPRT, differing in the 5'-attachment.

5' inversion is most likely to be achieved by a variant of TPRT called twin priming [14-16] (Figure 1). In twin priming, one DNA end may be annealed to the 3'-terminus of L1 RNA as in standard retrotransposition, whereas the other end is annealed to the same L1 RNA internally. As the results of annealing, nucleotides derived from either the target DNA or L1 cDNA, called microhomologies (MHs), are often observed at the 5'- and the 3'-ends of 5'-inverted L1 copies. After reverse transcription, L1 RNA could be degraded and the two cDNA strands are annealed at a region of short complementarity. This annealing was presumed on the basis of MHs at the inversion junction.

Template jumping, proposed from the analysis of another non-LTR retrotransposon family R2 [17-20], is a most likely mechanism for the generation of full-length L1 copies [21] (Figure 1). In the template jumping model, the RT molecule synthesizing first-strand cDNA jumps to the 3'-end of the target DNA in order to continue DNA polymerization.

The mechanism for 5' integration of 5'-truncated L1 copies has long been debated and various mechanisms for the 5' attachment have been proposed (Figure 1). Template jumping could contribute to 5'-truncated retrotransposition as well as to full-length retrotransposition [19,21]. The double TPRT model proposed that annealing of the internal sequence of cDNA with the 5' target site terminates reverse transcription and enhances second strand cDNA synthesis [15,22,23]. Reverse transcription termination before the completion could generate a Y-shaped intermediate, which could be healed by cellular DNA repair mechanisms, including microhomology-mediated end-joining (MMEJ) and non-homologous end-joining (NHEJ) [16,24-26].

Proteins encoded by L1, as well as other LINEs contribute to short interspersed nuclear element (SINE) mobilization [27,28]. There are two active SINE families, *Alu *and SVA, in the human genome [1,29]. *Alu *can be mobilized by L1 ORF2 proteins in the absence of L1 ORF1p [27,30]. This indicates that the retrotransposition mechanisms of L1 and *Alu *are not completely identical. SVA is a composite SINE containing fragments derived from *Alu *and human endogenous retrovirus (HERV)-K10 [29,31,32]; it is also considered to be mobilized depending on the L1 machinery.

Human L1 and *Alu *are usually flanked by ~15-bp TSDs [9,33,34]. TSDs are used to determine the junctions of L1 insertions in genome-wide screening [9]. However, in the case of short TSDs, an accidental sequence match covers true TSDs. Target analysis of nested transposons (TANT) is a method to characterize LINE insertions with short TSDs. It is, however, limited by the low copy number of insertions nested in other transposons [34-36]. The advantage of this method is its ability to assume pre-inserted empty sites. Pre-inserted sequences can also be characterized by analysing orthologous sequences of outgroup species.

In this study, human-specific insertions and chimpanzee-specific insertions of L1, *Alu*, SVA and non-coding RNA (ncRNA) retrocopies were characterized by a newly developed method that can distinguish insertions from deletions and can characterize insertions with short TSDs. Only 5'-truncated L1 insertion is associated with short TSDs. Different target site structures were found on 5'-truncated retrotranspositions in *cis *and retrotranspositions in *trans*.

## Results

### Precise characterization of retrotransposon insertions with extranucleotides and/or flanked by short TSDs

In this study, 6197 human-specific insertions and 2910 chimpanzee-specific insertions which satisfied all the following criteria were found: (1) length between 50 bp and 10,000 bp; (2) ≥5-bp direct repeats at both junctions; (3) not a part of segmental duplications; (4) not inserted in repetitive sequences; and (5) orthologous locus can be determined in either orangutan or macaque. Criteria 1-3 were applied in order to enrich the insertions of LINEs and SINEs. Criterion 4 was applied because the orthologous locus in orangutans and macaques could not be characterized for insertions inside of repetitive sequences. Criterion 5 was used to distinguish species-specific insertions from species-specific deletions.

Two primate genomes were used as outgroups to characterize the pre-inserted empty sites for each insertion; the orangutan and macaque genomes were complementary. Although empty sites for 3927 human-specific insertions and for 1281 chimpanzee-specific insertions were identified in the genomes of both species, empty sites for 2270 human-specific insertions and for 1629 chimpanzee-specific insertions were found only in the genome of either orangutan or macaque.

Since there are many studies of insertions of L1, *Alu *and SVA [3,9,15,16,21,23-25,27,37-39], new findings by the new method will be described in this report.

### Repertories of human-specific insertions and chimpanzee-specific insertions

Most of the species-specific insertions were derived from L1, *Alu *and SVA retrotransposition (Table 1). LTR retrotransposons, retrotransposed sequences (retrocopies) of mRNA and ncRNAs were also observed. mRNA retrocopies have been reported elsewhere [40].

**Table 1:** Human-specific and chimpanzee-specific insertions with ≥5bp TSDs

	Human-specific	Chimp-specific
Total	6197	2910
L1	1120	948
*Alu*	4197	1357
SVA	368	109
HERV and solo LTR	63	41
mRNA retrocopy	48	95
ncRNA retrocopy	14	13
DNA transposon	0	0
Uncharacterized*	387	347

Uncharacterized includes elements with nested insertion, internal deletion, internal duplication, 3' truncation, and template switch.

L1, long interspersed nuclear element-1; HERV, human endogenous retrovirus; LTR, long terminal repeat; mRNA, messenger RNA; ncRNA, non-coding RNA.

The origins of the ncRNA retrocopies were ribosomal RNA (rRNA), small nuclear RNA (snRNA), small nucleolar RNA (snoRNA), transfer RNA (tRNA), 7SK RNA, and 7SL RNA (Additional File 1). Among 25 ncRNA retrocopies, only three contained polyA tails. These three could be retrocopies generated by template switching during the very beginning of reverse transcription [41,42]. The remaining 22 retrocopies were usually 3'-truncated and similar to the tailless retropseudogenes reported by Schmitz *et al*. [43]. Tailless retropseudogenes were originally reported as retrocopies of tRNA that are 3'-truncated, without A-rich tails, and flanked by TSDs. They also detected tailless retrocopies of rRNAs and snRNAs. Tailless retrocopies of snoRNAs, 7SK RNA and 7SL RNA are reported for the first time here, indicating that all ncRNAs can become templates of tailless retrocopies.

### Frequent existence of 5' extranucleotides in full-length L1 retrotransposition

The 5'-terminal heterogeneity of L1 was first analysed because the insertion junction could be determined without ambiguity. Among the factors causing heterogeneous 5' ends of L1 [44,45], Yin Yang 1 (YY1) transcription factor binding to L1 nucleotides 12-22 (CAAGATGGCCG) could direct transcription to initiate at heterogeneous purine residues located near the 5'-end of L1.

**Figure 2:**
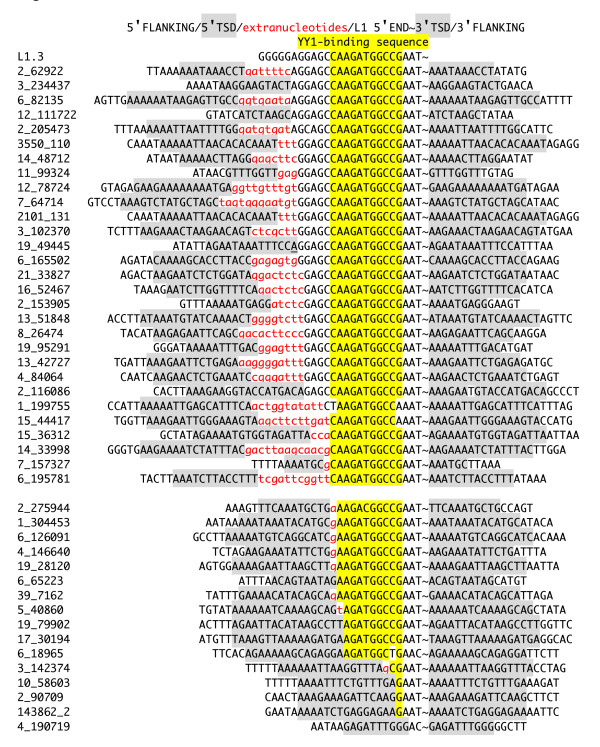
**Junction sequences of L1 insertions starting between positions 6-25**. Target site duplications are shaded in grey; microhomologies are underlined; 5' extranucleotides are colored in red and in lowercases; and YY1-binding sequences are shaded in yellow.

The frequent existence of stretches of 5' extranucleotides for insertions with the YY1-binding sequence was a remarkable difference between the insertions with and those without a complete YY1-binding sequence (Figure 2, red). Hereafter, the elements including a complete YY1-binding sequence were termed full-length elements. Most stretches of extranucleotides flanked by full-length L1 are likely derived from transcription initiation upstream from the L1 sequence [44].

### 5' inversion occurred frequently with L1 retrotransposition but rarely with *Alu *and SVA retrotransposition

Full-length L1 insertions occupied 16.2% of all the L1 insertions flanked by 5-30 bp TSDs (Table 2). Full-length L1 insertions were extremely less frequent in the chimpanzee genome than in the human genome, consistent with the previous study [38]. The most abundant type of L1 insertion was 5'-truncated, which occupied more than a half of all L1 insertions. The 5'-inverted L1 insertions occupied ~30% of both the human- and the chimpanzee-specific L1 insertions, and it is consistent with the previous study by Ostertag and Kazazian [14].

**Table 2:** Three types of L1 insertions in human and chimpanzee

	Human	Chimp	Total
Full-length	286 (25.5%)	49 (5.2%)	335 (16.2%)
5'-truncated	490 (43.8%)	604 (63.7%)	1094 (52.9%)
5'-inverted	344 (30.7%)	295 (31.1%)	639 (30.9%)
Total	1120	948	2068

In sharp contrast, most of the *Alu *insertions were full length even with the strict definition in which only *Alu *insertions starting with the first nucleotide were considered to be full length (Table 3). Only one example of a 5'-inverted *Alu *insertion was found in this study (Additional File 2) showing a structure similar to inverted L1 and mRNA retrocopies [14-16,40]. The quite different copy numbers of human- and chimpanzee-specific *Alu *insertions have already been reported by Mills *et al*. [37] and are confirmed by the present study.

**Table 3:** Three types of *Alu *insertions in human and chimpanzee

	Human	Chimp	Total
Full-length	3449 (82.2%)	1023 (75.4%)	4472 (80.5%)
5'-truncated	748 (17.8%)	333 (24.5%)	1081 (19.5%)
5'-inverted	0 (0%)	1 (0.1%)	1 (0.0%)
Total	4197	1357	5554

As the 5'-end of SVA is composed of variable numbers of degenerate CCCTCT repeats [29], full-length and 5'-truncated SVAs could not be distinguished correctly. Among 477 SVA insertions, 95 started with G followed by degenerated CCCTCT repeats. Some of them are likely to be full-length insertions with a reverse-transcribed 5'-cap, like full-length L1 insertions [16]. Only four instances of 5'-inverted SVA insertions were found (Additional File 2). The 5'-inverted SVA insertion showed a structure similar to inverted L1, mRNA retrocopies and *Alu*. The proportion of the 5'-inverted SVA (4/477 = 0.84%) was very small.

### 5'-inverted L1 was rarely inserted with extranucleotides at the 5' junctions

5'-inverted L1 was less frequently inserted with extranucleotides at the 5' junctions than 5'-truncated L1, 5'-truncated *Alu*, and full-length *Alu *(Table 4). The frequency of 5'-inverted L1 with extranucleotides was between those in the bioinformatics study and the retrotransposition assay in HeLa cells by Ichiyanagi *et al*. [35]. There was no difference between full-length and 5'-truncated *Alu *insertions in terms of the frequency of 5' extranucleotides.

**Table 4:** The frequency of insertions with 5' extranucleotides

Type of insertion	No. of insertions with extranucleotides	Frequency
5'-inverted L1	25	3.9%
5'-truncated L1	160	14.6%
5'-truncated *Alu*	126	11.7%
Full-length *Alu*	554	12.4%

### 5'-truncated L1 copies were sometimes accompanied by short TSDs

**Figure 3:**
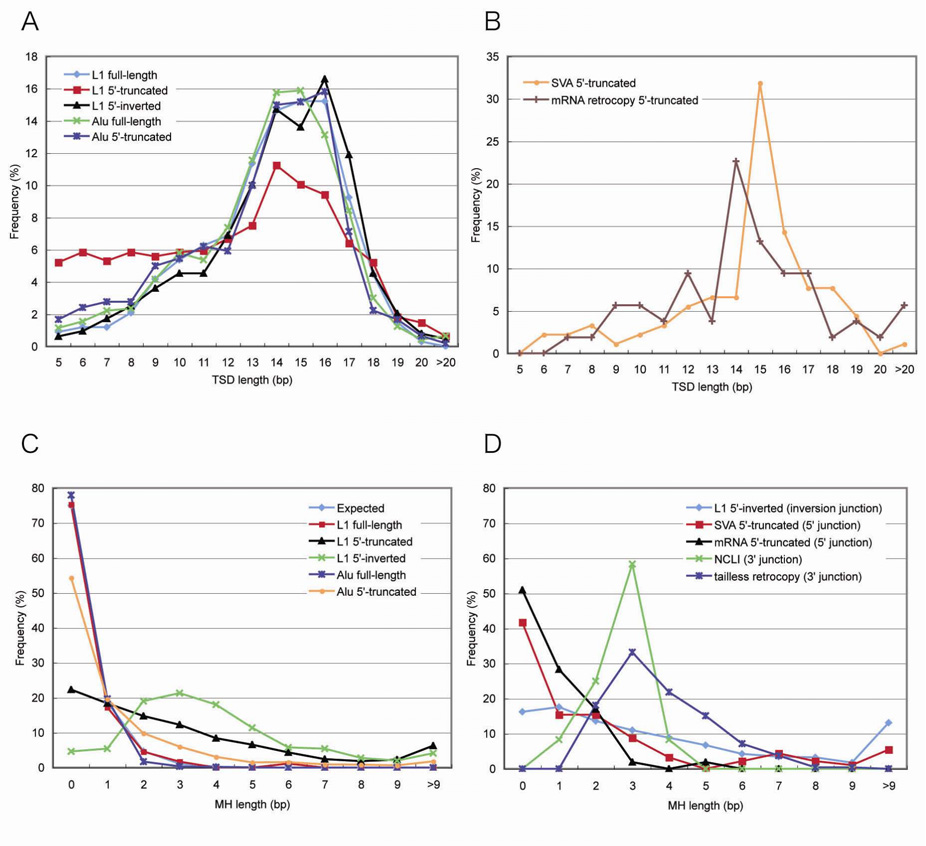
**Target site duplication (TSD) and mircohomogous (MH) length distribution**. (A) TSD length distribution of L1 and *Alu*. (B) TSD length distribution of SVA and messenger RNA retrocopy. (C) Length distribution of MH at 5' junctions of L1 and *Alu*. (D) Length distribution of MH at inversion junction of 5'-inverted L1, 5' junctions of SVA and mRNA retrocopies and 3' junctions of non-canonical L1 insertion (NCLI) and transfer RNA tailless retrocopies.

The distributions of TSD length in full-length L1, 5'-truncated L1, 5'-inverted L1, full-length *Alu *and 5'-truncated *Alu *were analysed independently (Figure 3A). The human- and chimpanzee-specific insertions showed a nearly identical TSD distribution (data not shown) and, therefore, their insertions were combined for further analysis.

The TSD length distribution of full-length L1 was similar to that considered true for L1. The peak length was 14-16 bp, and there was a small number of L1 with short TSDs (Figure 3A). These results support the ability of the analysis to characterize insertions with short TSD length precisely.

The relatively frequent observation of short TSDs in 5'-truncated L1 was remarkable. In contrast, the TSD length distribution between full-length and 5'-inverted L1 insertions was similar. To investigate whether the TSD length distribution is different among the types of insertions, the chi-square test was performed (Table 5). Short TSDs were significantly more abundant in 5'-truncated L1 insertions than in full-length L1, 5'-inverted L1, full-length *Alu*, and 5'-truncated *Alu *insertions.

**Table 5:** Chi-square or Fisher's exact test for the frequency of ≤ 9-bp target site duplications

Comparison	*P*- value
5'-truncated L1 > full-length L1	5.7e-12***
5'-truncated L1 > 5'-inverted L1	1.2e-19***
5'-truncated L1 > full-length *Alu*	5.3e-43***
5'-truncated L1 > 5'-truncated *Alu*	5.9e-14***
5'-truncated L1 > 5'-truncated SVA	7.7e-5***
5'-truncated L1 > 5'-truncated mRNA retrocopy	0.0038**

** *P*- value < 0.01

*** *P*- value < 0.001

### Short TSDs observed in 5'-truncated L1 copies were likely dependent on endonuclease

**Figure 4:**
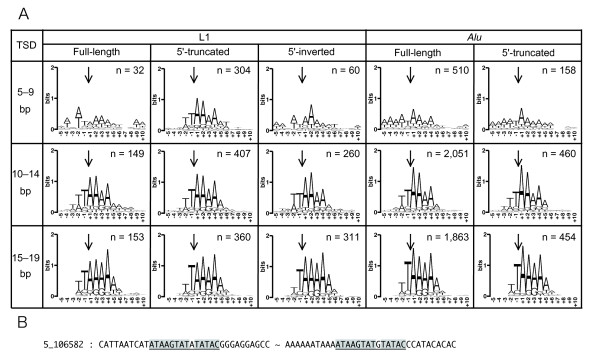
**Insertion site sequences**. (A) Target sequence logos. Arrow indicates the position of 3' insertion site. Sample numbers are represented at upper right. As insertions with 20-bp target site duplications (TSDs) are not included, the sums of sample numbers are not equal to those in Tables 2 and 3. (B) An example of short TSDs that were likely to be parts of long TSDs including substitution. TSDs are underlined and identical sequences in TSDs are shaded in black.

It was examined whether 5'-truncated L1 insertions with short TSDs are generated by endonuclease-independent retrotransposition. Sequence logos [46] of target sequences were created for each type of insertions at each 5-bp TSD length (Figure 4A). Target sequence preference for TT/AAAA could be seen for all types of insertions with ≥10-bp TSDs, indicating L1 EN-dependent retrotransposition. In contrast, only 5'-truncated L1 insertions showed the sequence preference for TT/AAAA when flanked with ≤ 9-bp TSDs. In this study, TSDs were defined as direct repeats of ≥5-bp perfect sequence match, and therefore, long TSDs with substitutions could not be detected and were annotated as short TSDs instead.

On reinvestigating TSDs in a relaxed TSD definition for L1 insertions flanked by 5-bp and 6-bp perfect-match TSDs (Figure 4B, Table 6, Additional Files 3 and 4), most of the short TSDs of L1 and *Alu *insertions were extendable if a 1-bp nucleotide substitution sandwiched by ≥2-bp stretches of homologous nucleotides was allowed in the TSDs. In a relaxed TSD definition, the target sequences with short TSDs of insertions of full-length L1, 5'-inverted L1, full-length *Alu *and 5'-truncated *Alu *insertions agreed better with TT/AAAA (Additional Files 3 and 4). Therefore, the actual TSDs were likely to include substitutions. On the contrary, less than half of the 5'-truncated L1 insertions could be extended. This finding and the fact that 5'-truncated L1 insertions with short TSDs showed target sequence preference for TT/AAAA even in a strict TSD definition indicated that 5'-truncated L1 insertions were actually accompanied by short TSDs and that short TSDs were generated by an L1 EN-dependent machinery.

**Table 6:** Short target site duplications (TSDs) with or without allowing nucleotide substitutions

	L1			*Alu*	
	
	Full	Trun	Inv	Full	Trun
5-bp TSD in relaxed criterion*/ 5-bp TSD in strict criterion*	0/3 (0%)	36/56 (64%)	0/4 (0%)	13/51 (25%)	5/18 (28%)
6-bp TSD in relaxed criterion*/ 6-bp TSD in strict criterion*	0/4 (0%)	51/64 (80%)	0/6 (0%)	14/69 (20%)	6/26 (23%)

*TSD in relaxed criterion allows 1-bp substitution sandwiched by ≥2-bp homologous nucleotides, but TSD in strict criterion does not.

Full, full-length; Trun, 5'-truncated; Inv, 5'-inverted.

### MH length distribution at 5' junctions

Next 5' MHs were analysed. Full-length L1 insertions showed MH length distribution predicted as if each of four nucleotides was observed by chance (Figure 3C), consistent with the previous study [25].

5'-inverted L1 insertions were clearly dependent on MHs; only 28 of 605 (4.6%) 5'-inverted L1 insertions were free from MHs. Approximately 70% showed MHs of 2-5 bp. Nearly 80% of all the 5'-truncated L1 insertions were also associated with MHs, but the MH length distributions of these insertions were distinct from that of the 5'-inverted L1 insertions; 5'-truncated L1 appeared less dependent on MHs than 5'-inverted L1. The chi-square test supported the different dependency (Table 7). The frequency of MHs was in the order full-length L1 < 5'-truncated L1 < 5'-inverted L1.

**Table 7:** Chi-square or Fisher's exact test for the frequency of ≥1-bp microhomologies (MHs)

Comparison	*P*-value
5'-inverted L1 > 5'-truncated L1	5.9e-21***
5'-truncated L1 > Full-length L1	1.1e-43***
5'-truncated L1 > 5'-truncated *Alu*	9.8e-46***
5' junction of 5'-inverted L1 > Inversion junction of 5'-inverted L1	3.6e-11***
Inversion junction of 5'-inverted L1 > 5' junction of full-length L1	2.3e-51***
5'-truncated L1 > 5'-truncated SVA	2.3e-5***
5'-truncated L1 > 5'-truncated mRNA retrocopies	3.7e-6***

*** *P*-value < 0.001

Full-length *Alu *insertions were similar to full-length L1 insertions in terms of the frequency of MHs (Figure 3C). 5'-truncated *Alu *showed a weak dependence on MHs. However, the frequency of MHs in 5'-truncated *Alu *was not as high as that in 5'-truncated L1 (Table 7). In summary, the frequency of MHs was in the order full-length L1 ≈ full-length *Alu *< 5'-truncated *Alu *< 5'-truncated L1 < 5'-inverted L1.

### MH length distribution of inversion junctions and 3' junctions

The MH length distribution at the inversion junction of 5'-inverted L1 was similar to that at the 5' junction of 5'-truncated L1 (Figure 3D). The frequency of insertions without MHs at the inversion junction of 5'-inverted L1 was higher than that at the 5' junction of 5'-inverted L1 and lower than that at the 5' junction of full-length L1 (Table 7).

The MH length distribution at the 3' junctions of endonuclease-independent L1 retrotransposition [47] and tailless retrocopies of tRNAs [43] was investigated (Figure 3D). The distributions of 3' MHs of endonuclease-independent L1 retrotransposition and tailless retrocopies were similar to those of the 5' MHs of 5'-inverted L1, especially in terms of the frequency of short MHs. The large peak at 3 bp in the 3' junctions of endonuclease-independent L1 insertions is likely due to its small data set.

### SVA and mRNA retrocopy showed similar length distribution of TSDs and MHs as *Alu*

Figure 3B and 3D show the distributions of TSDs and MHs at the 5' junctions of 5'-truncated SVA and 5'-truncated mRNA retrocopy insertions. It is clear that short TSDs were rarely associated with insertions of 5'-truncated SVA and 5'-truncated mRNA retrocopies (Figure 3B). Because of the small sample sizes, Fisher's exact test, not chi-square test, was used to show statistical significance for the difference (Table 5). The order of frequency of insertion with ≤ 9-bp TSDs were 5'-truncated L1 > 5'-truncated SVA ≈ 5'-truncated mRNA retrocopy. The frequency of insertions without MHs was higher for the 5'-truncated SVA and 5'-truncated mRNA retrocopies than for 5'-truncated L1 (Figure 3D; Table 7). In summary, the length distributions of both TSDs and MHs associated with insertions of 5'-truncated SVA and 5'-truncated mRNA retrocopies were similar to those associated with 5'-truncated *Alu *insertions and dissimilar to those associated with 5'-truncated L1.

### No correlation between TSD length and MHs

As the length distributions of TSDs and MHs are distinguishable features between retrotransposition in *cis *and retrotransposition in *trans*, one can speculate that short TSDs are dependent on the long MHs. However, there was no correlation between TSD length and the existence of MHs (data not shown).

## Discussion

### Short TSDs specifically associated with 5'-truncated L1

The most significant finding in this study was that a bias towards shorter TSDs in truncated L1s, although insertions with longer TSDs still predominate even in the 5'-truncated elements. The TSD length distribution of 5'-truncated L1 was different from those of full-length and 5'-inverted L1. In the retrotransposition assay in HeLa cells performed by Gilbert *et al*. [16], all 6 full-length and 17 5'-inverted L1 insertions were flanked with TSDs of 10-17 bp, whereas 5'-truncated L1 insertions were frequently coupled with radical alteration of the target sites, including extremely long TSDs, target site deletions, and short TSDs of < 10 bp. These radical alterations were considered to be due to peculiarities associated with HeLa cells, but the present analysis showed that at least short TSDs are actually coupled with 5'-truncated L1 insertions *in vivo*. TSDs only between 5 bp and 30 bp in length were collected in this study, and further study to characterize shorter and longer TSDs is needed to clarify other radical alterations *in vivo*.

### Difference in 5'-truncated insertion between in *cis *and in *trans*

5'-truncated retrotransposition in *trans *is not associated with short TSDs. What causes the difference between retrotransposition in *cis *and retrotransposition in *trans*? There have been many papers describing the differences between L1 and *Alu *retrotransposition [27,30,48]. However, SVA and mRNA retrocopies showed TSD length distributions similar to *Alu*. Therefore, the binding ability to signal recognition particles [27] and the type of RNA polymerase [48] can be excluded from the answer to this question.

It is difficult to propose a model from the data described here, but one possible explanation is that the longer time for reverse transcription of L1 RNA, longer than other RNAs, would attract cellular DNA repair machineries. Gilbert *et al*. [16] proposed abortive retrotransposition generates Y-branched intermediate and it is probably healed by microhomology-mediated end joining to generate the radical alteration of insertion sites. When the top strand is cleaved before the completion of first-strand cDNA synthesis, the L1 retrotransposition intermediate constitutes a DNA double-strand break [49]. From the observation of few 5' inversion in *trans*-mobilized elements, top strand cleavage before the completion of first-strand cDNA synthesis could be nearly an exclusive feature of L1 retrotransposition [14] and possibly increases the 5' rejoining by cellular DNA repair system.

### Technical improvements

In this study, large-scale identification of retrotransposon insertions was achieved. Only closely related genomic sequences and at least one genomic sequence as an obvious outgroup were needed for analysis. One important improvement is that insertions could be distinguished from deletions, enabling the characterization of insertions with no signatures, and with drastic rearrangements.

The TSD length analysed in this study was restricted between 5 and 30 bp because of the limit of the computational power and manual adjustment, but all insertions with or without TSDs could be characterized in the method developed in this study. TSDs of L1 and *trans*-mobilized elements by L1 are relatively long among the transposable elements. Many non-LTR retrotransposons show shorter TSDs [36], DNA transposons and LTR retrotransposons show much shorter TSDs, and Helitrons show no TSDs [50]. The method used in this study has the power to characterize these elements systematically and independently of TSD length.

## Conclusions

The newly developed method using outgroup genome sequences can distinguish insertions from deletions and can characterize insertions with short TSDs correctly. This method can be applied to characterize elements without any obvious boundary structures. This study confirmed the present knowledge of L1-dependent retrotransposition, and revealed the relatively frequent appearance of short TSDs coupled with 5'-truncated L1 *in vivo*.

## Materials and methods

### Genome sequences

Genome sequences of human, common chimpanzee, Sumatran orangutan, and rhesus macaque and the pairwise alignment between human and chimpanzee genomes were downloaded from the University of California, Santa Cruz Genome Browser website [51].

### Characterization of species-specific insertions and TSDs

Human- and chimpanzee-specific insertions were collected as described previously [40]. L1, *Alu *and SVA insertions were characterized by using RepeatMasker [52] with a library including only three human repetitive sequences: L1.3 (DDBJ/EMBL/Genbank accession number: L19088), *Alu*Y [53] and SVA [53]. RepeatMasker was also run with the repeat library downloaded from RepBase [53] in order to characterize ncRNAs and endogenous retroviruses. Insertions including more than two types of insertions were manually excluded, with the exception of insertions in which 3'-transduced sequences could be characterized on the basis of large sequence variation from the consensus. Insertions with nested insertions, deletions, or duplications inside of the elements were also excluded.

### Analysis of target sequences

Sequence logos of the target sequences were generated by using WebLogo [46].

### Analysis of MH length

To identify MHs at 5' junctions, RepeatMasker was used with two different queries; one included only sequences of inserts and the other included sequences of both inserts and TSDs. If repetitive elements were extended into TSDs, the extended sequences were considered to be MHs. Nucleotides corresponding to both inverted and noninverted segments of repetitive elements were counted as MHs at inversion junctions, and the MHs at 3' junctions were manually counted in the published data sets [43,47]. Sen *et al*. [47] reported 11 3' junction sequences of endonuclease-independent L1 insertions, and Schmitz *et al*. [43] reported 238 3' junction sequences of tailless retrocopies of tRNA.

*bona fide *5'-truncated SVA insertions were obtained by collecting SVA insertions without CCCTCT repeats. Consequently, 91 5'-truncated SVA insertions were found. In the case of mRNA retrocopies, because the transcription start site is considered to be heterogeneous, the retrocopies starting at > 50 bp downstream from the +1 position of annotated transcripts were considered as 5'-truncated mRNA retrocopies, and 53 5'-truncated retrocopies were identified.

## Abbreviations

EN: endonuclease; HERV: human endogenous retrovirus; LINE: long interspersed nuclear element; L1: LINE-1; LTR: long terminal repeat; MH: microhomology; MMEJ: microhomology-mediated end joining; NHEJ: nonhomologous end joining; ORF1p: open reading frame 1 protein; ORF2p: open reading frame 2 protein; RT: reverse transcriptase; SINE: short interspersed nuclear element; TPRT: target-primed reverse transcription; TSD: target site duplication; TANT: target analysis of nested transposons.

## Competing interests

The author declares that he has no competing interests.

## Supplementary Material

Additional file 1 Non-coding RNA retrocopies. Click here for file

Additional file 2 Structures of 5'-inverted *Alu *and SVA. Click here for file

Additional file 3 Sequences around 5-bp assumed target site duplications. Click here for file

Additional file 4 Sequences around 6-bp assumed target site duplications. Click here for file
