# Supplementary material for: Different integration site structures between L1 protein-mediated retrotransposition in cis and retrotransposition in trans
Source: Mob DNA. 2010 Jul 8;1:17. doi: 10.1186/1759-8753-1-17 (PMC2912911; doi:10.1186/1759-8753-1-17)
Supplement: Additional file 1 — Non-coding RNA retrocopies. [file 1759-8753-1-17-S1.PDF]

Additional file 1. ncRNA retrocopies.

	Human-specific	Chimpanzee-specific
rRNA		
5S rRNA	5	2
snRNA		
U2 snRNA	1	1
U5 snRNA	0	2
U6 snRNA	2	0
snoRNA		
U3 snoRNA	1	0
U13 snoRNA	0	1
HBII-85 snoRNA	0	1
tRNAs	4	2
7SK RNA	1	3
7SL RNA	0	1
