# Supplementary material for: Different integration site structures between L1 protein-mediated retrotransposition in cis and retrotransposition in trans
Source: Mob DNA. 2010 Jul 8;1:17. doi: 10.1186/1759-8753-1-17 (PMC2912911; doi:10.1186/1759-8753-1-17)
Supplement: Additional file 2 — Structures of 5'-inverted Alu and SVA. [file 1759-8753-1-17-S2.PDF]

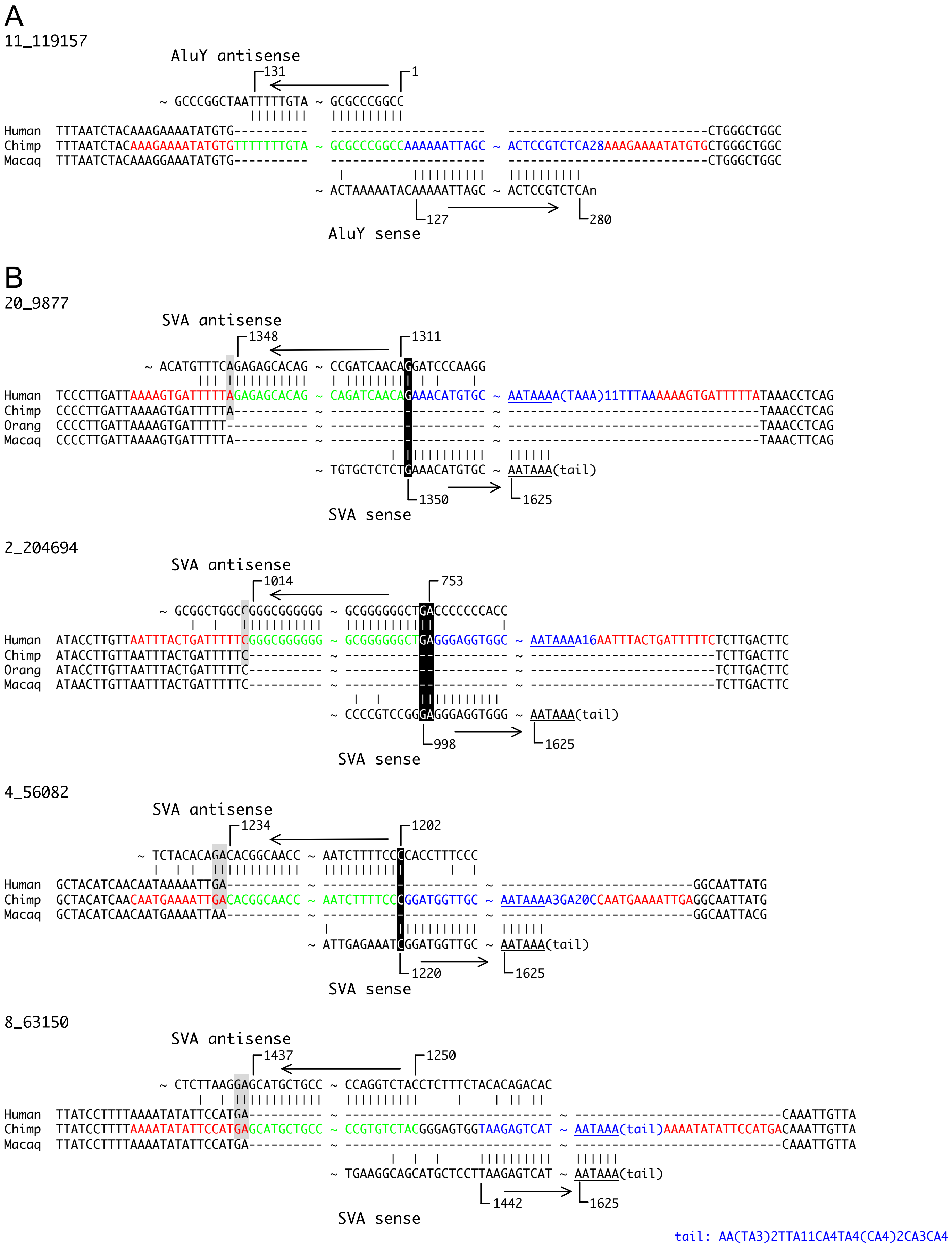

Additional file 2. Structures of 5'-inverted *Alu* (A) and SVA (B). Genome sequences of human,

chimpanzee (chimp), orangutan (orang) and rhesus macaque (macaq) are aligned with RNA sense and antisense sequences. TSD sequences are in red. MHs at 5' junction are shaded, while MHs at inversion junction are highlighted. Nucleotides in inverted segment are in green, whereas those in noninverted segment are in blue. Polyadenylation signals are underlined. Numbers of RNA sense and antisense sequences indicate positions in respective RNA.
