# Supplementary material for: Different integration site structures between L1 protein-mediated retrotransposition in cis and retrotransposition in trans
Source: Mob DNA. 2010 Jul 8;1:17. doi: 10.1186/1759-8753-1-17 (PMC2912911; doi:10.1186/1759-8753-1-17)
Supplement: Additional file 3 — Sequences around 5-bp assumed target site duplications. [file 1759-8753-1-17-S3.PDF]

L1, full-length, 3 sequences.

```
Name        : Sequence around 5' junction                   | Sequence around 3' junction
6_34700     : aaatagAtgtAAAgAAAATaTTAAAgggggGAggagccAaGatgG | tcccccAaaaAAAtAAAATcTTAAAaataaGAtctcatAtGcaaG
5_106582    : AcAttAATcAtATAAGTATaTATACgggaggAgcCAAgatgGccg | AaAaaAATaAaATAAGTATgTATACccatacAcaCAAaccaGtaa
3_180780    : ttgAcAtAAcAgAAAAcGTaCCATGggaggAgcCAaGaTggccgA | aaaAaAaAAaAaAAAAaGTgCCATGttccaAtgCAcGtTtctacA
```

L1, 5’-truncated, 56 sequences.

```
Name        : Sequence around 5' junction                   | Sequence around 3' junction
1_54586     : tcAaAatagctgtTAaAaAaATATGggaAggggaaTAtcaCactc | atAtAtatatataTAtAtAtATATGattAaaaactTAaatCgtgt
21_12106    : tAAaaTAtAcTATgtcccttAAAAAaACAccgCATAttctcacgc | aAActTAaAgTATaataaaaAAAAAtACAtgaCATAaatattatg
10_62164    : ttaccattaTtAtAtAttttAAAAGaactggAaatAcCaTTTGac | aagtataatTaAaAaAaaaaAAAAGgtactcAtgaAgCtTTTGgt
4_206535    : AAAtAtgcAgtgttAAGAAtGTTCAaaTacTatgcagCcataaAA | AAAaAaaaAaaaagAAGAAcGTTCAttTctTcatggtCatggtAA
10_183468   : acaAtgtAtgAAttggtcttAACAAacAgcaTggtactggtAcca | ttcAaaaAaaAAaaaaaaaaAACAAtgAagtTaccggcttaAggt
4_130851    : AAtGAAcAcgggctAttcttAGAAAcAGAAATAgcAtTgggagAt | AAaGAAaAgaaaaaAaaaaaAGAAAtAGAAATAcaAaTttttaAa
16_85891    : gAcAtgAAAcctgcAAAtAcAGAGAaGACatTtaTgcaGccAaaa | tAtAatAAAaaaaaAAAaAaAGAGAtGACtgTacTtttGaaAtgc
7_40095     : TggcAgtAAgtttgggAgAtAAGAAcGGGCAGAgaagttcaTgtc | TtaaAaaAAaaaaaaaAaAaAAGAAtGGGCAGAagttcatcTtat
4_102289    : ttcaaTttggttctAttttcAAAACataggcgtGggcaaggActt | caattTaaaaaaaaAaaaaaAAAACtgcttacaGtttttcaAgca
1_175174    : gtgcaacaaATaAAgAcAAcAAAAAcAaTtgtggtGgGgtcgggg | agattcactATtAAaAaAAaAAAAAaAtTgagataGtGaaaataa
766_39      : TAgagccttActttAAGAAaGTTAAcaAtgaacTcaaAcaaATTT | TAatttaaaAaaaaAAGAAtGTTAAacAactttTgtgAatgATTT
7_42549     : tcttAtcAAtAAATAAGTAaAATCAcaCaTggaCacAggAagggg | aaaaAaaAAaAAATAAGTAtAATCAacCtTtctCtaAatAtatct
3_230407    : tagccAttttAgAgAtgtttGAAAAcacTgTTggTgGgacTgtaa | ataaaAaaaaAaAaAaaaawaGAAAAtttTcTTtaTtGcttTcatt
3_134902    : AtttcaTAtgtgActAcAttAAGACcagcatgGcACatgTaTacA | AaacatTAaaaaAaaAaAaaAAGACatttcatGtACtaaTcTctA
4_31847     : TaAActTAgAgaAtAAAAAcAATCGcagccATaaAAaaTgAtgag | TtAAagTAtAatAaAAAAAaAATCGatcatATtgAAgtTcAgttt
3_107067    : tgcaggctAtggAAgAgcAtGAAAGcAggaaggggaacAtcAcAc | ctgtaaaaAaaaAAaAaaAaGAAAGtAtagtttatgtaAggAaAa
15_117419   : ttGTgTttTggtttcccttttAAGAAcgcGTtagtgggtgcAgcgC | aaGTaTaaTaaaaaaaaaaaAAGAAgagGTagaaaaaataAaatC
18_55064    : AcAtAttAgtgctAtAAAAtATTAActAAcCTgcACAAtgTgcaC | AaAaAaaAaaaaaAaAAAAaATTAAagAAtCTctACAAaaTatgC
8_31719     : tgtcAccAAAAgActctattAAGAAcTataaAgAcAcatgcacac | aagtAtaAAAAaAaaaagaaAAGAAaTtgctAcAgAacgttcttt
6_163851    : AAactcAcAcAtAtcAtgAtAAGTGcTgctaTaaagacacatgca | AAtaaaAaAaAgAaaAaaAaAAGTGtTtgctTcttatgttcctgc
5_31060     : tgAacccAAcaGaTGTtAAgAAGAAgAgttcaTgtccTTtgtagg | aaAgaaaAAggGtTGTaAAaAAGAAaAtggatTtcatTTgaggaa
2_192949    : ctgTtAtgAAAgAcAcAtgtAAAACgggcaaggActTcAtgTccA | taaTaAaaAAAaAaAaAaaaAAAACacaaggatAaaTaAgtTgaA
18262_38    : tTgAAgcaggggAgtgacttAAAACtAGGTGGTggAtatAccTaa | aTtAAaacaaaaAaaacaaaAAAACaAGGTGGTatAagaAagTgc
17_50365    : AttatAttAggGgActttttAAAAAtgtaTAcgTAtgTaactAac | AaagaAagAaaGaAagaaaaAAAAAgtctTAatTAaaTggagAga
10_115520   : cAAttaccAAtAAAcAttTgAAAATttacCAtTcagGAcataggc | aAAaacttAAaAAAaAaaTaAAAATaattCAcTagtGAttacaat
21_6833     : AAcaTtagAattAgtcAcAtAAAAGaaAtaggaAcacttttacac | AAagTataAtaaAaaaAaAaAAAAGtcAagaacAagaccaagatg
3_176961    : gccAgatggAgAtactgctaAATAAaAatgAtGAgtTCAtaTcct | ataAttaaaAaAataataatAATAAtAtccAaGAtaTCAggTttc
5_202496    : tgTtaCAgAccAtAttAtttAAGAAaatgTggcacatatAcAcca | atTctCAaAaaAaAaaAaagAAGAAgtaaTcattgggtaAgAgtc
4_148622    : gtAcAtcagAgAgAgcAAaaCTGTGacaccaTCaTtctcaGtaaa | aaAaAaacaAaAaAaaAAccCTGTGctttagTCtTctcttGggtg
3_128852    : gggtctccActtggAcctttAGAAGtTTCTTCAAgAaaTagGaac | aaaaaaaaAaaaaaAaaaaaAGAAGcTTCTTCAAcActTgaGtgg
2_99331     : tttgttCTgAAggtttgtAtAAAGGagaCacttCtCAaaaGaagA | aaaaaaCTaAAaaaaaaaAaAAAGGtatCcaccCaCAtctGggcA
14_84164    : aAtAgctTggctttAcAAAgAAACAaggggaatAtcacActctgg | tAaAaaaTtaaaaaAaAAAaAAACAttatcctcAgggaAggaatc
14_87831    : aagTCaTggcAgAcAtccctAAAAAcaccgcATaTTctCActcAt | ctcTCcTctgAaAaAaaaaaAAAAAagtataATtTTgCAaagAc
3_233048    : AgAtgAttcAAAaTgctTttAAAAAgcacTtcTcAaaagaagacA | AtAaaAaaaAAAtTaaaTaaAAAAAtagaTatTaAtggagttgaA
6_166675    : tgAAgccAggatgtGAAATgGTTTTtttTcAgccatAAaAAatga | aaAAaaaAaagaaaGAAATtGTTTTaaaTgAatggaAAgAAcact
3_162040    : ccacttgGAActcAtcggttAGAAAcaggaaggggaaTatcacac | aagaaaaGAAaaaAaaaaaaAGAAAtgtatcaccttgTccacttt
2_135094    : ctgtgcttAttttAGAAAAtAAAACcAtaAaAaaTgAtGagTtca | aaaaaaaaAaaaaAGAAAAgAAAACaAatAtAttTtAaGgtTatt
9_60257     : tTAAatcAAtAAAtctctttAAGTTcataTgtaacTaaCcTgCac | aTAAtaaAAaAAAaaagaaaAAGTTtcctTtattgTgtCtTcCtt
28223_13    : aAtAcggaaAAGacagtaaaAAAAAtgtgtGgAAagAacATggaa | tAgAactttAAGtataatttAAAAAgtgtgGaAAgaAtaATatgg
3_134113    : tacgTttTtATAtTTtAcAtAAAGAcaTggATgAaactggaaAcC | ataaTaaTaATAaTTaAaAaAAAGAatTttATtActgaaattAaC
7_132313    : AAAAtaAAAaTctcAAtgAaATATAcctaatgctagAtgAcgagt | AAAAatAAAtTtaaAAaaAtATATAatctcacaccaAgaAaacaa
16_93670    : AagaAttGTtgAAcctcttgAAATCctcAtaGgtGgGAattgAAc | ActtAaaGTatAAtaaaaaaAAATCtgtAacGtgGaGAtaacAAt
11_157503   : tggccccagAgcctgcAcAcAGAAGgaaacaaCaGgtgCTggagA | aaataaataAataaatAaAaAGAAGtcccgtgCtGtgcCTctccA
1_17564     : gcAggcctATtctgAcAttGAAAGgaAtacTatgcagcCatAAa | aaAataaaATaaaatAaAaaGAAAGttAacaTtgattctCccAAc
20_47485    : atTAAGgttAAAAAgAAAAcAACTAgaatcTaCaatgaactCaaa | taTAAGaaaAAAAAaAAAAaAACTAtgcatTcCtgcctcagCtgg
5_202194    : tgctAAAcAggcaAcccttcAAAAAgactggattaagAAaAtgTG | aataAAAaAtaatAataaaaAAAAActagtctaggttAAcAaaTG
4_175572    : tTcAtAcatagtaAccAtctAATAGggACTgtaaactagttCAAc | cTtAaAgtataatAaaAaaaAATAGatACTtcctgaatcaaCAAa
9_146068    : ATcAggaAttcctActtAAaCATGTggaTgaAAttggaAatcAtc | ATaAtttAaaaaaAaaaAAcCATGTaagTtgAAcctatAgagAaa
8_118088    : tctAcgcctAgttgccctttAAAACggcacatGtAtAcatatgtA | aaaAgaaaaAagaaaaaaaaAAAACatactgaGaAaAttctctaA
14645_72    : AagAcAttAttGAAAAGGCgTTGCCggcAcatgtcGAccTaTGTA | AtaAtAaaAaaGAAAAGGCcTTGCCtatAttcactGAtgTtTGTA
17_52643    : ctaAATAgAtctgtAtaaaaTTTTTtAgcAtTgggagaTataCct | tgtAATAaAaaaaaAattttTTTTTgAtaAaTcacctcTtatCtg
8_66471     : cTcAgAAgaggcAAATgActAAAGAtcaAaaCcaCAAtGAgatAc | aTaAaAAataaaAAATaAaaAAAGAaagAgtCttCAAaGActaAg
7_51036     : ctggggttggAtAgtAttttAAAGGaCgagtTAgtgggtgCagcg | taattaaaaaAaAaaAaaaaAAAGGtCcttaTAaaaaagaCgaaa
14_86423    : ctgttgatAttttAggtcAtTAAAGacGagttagtgGgtgcagcg | tataattaAaagaAaaaaAaTAAAGtaGgtaataatGtctgtcat
5_104141    : cATAtgTtgAgaAAActttaAAAAAcgagtTAgtGggtgcagcGc | tATAatTaaAatAAAtaaagAAAAAtacaaTAaaGaaaaagtaGa
17_70359    : AgccttAgttgAggGAccttAAGAAtTGgGagaTAtaCCtAaTgC | AaaaaaAaaaaAaaGAaagaAAGAAaTGaGttcTAggCCaAgTtC
```

L1, 5’-inverted, 4 sequences

```
Name        : Sequence around 5' junction                   | Sequence around 3' junction
9_59081     : taTgTgAtgttAcgtcAAAtAAAGAgCAATCAGCaccatTtATta | acTtTaAaaaaAaaaaAAAaAAAGAaCAATCAGCcaggcTaATcc
3_144163    : AttcagatgcaagaAcCCAcCTTCCagtacCatGCtgttTtg gG | AaaacttaaagtatAtCCAaCTTCCtcctgCtaGCaagcTattaG
7_74931     : cttcAtcAAGATATAGGAAgAAATAttTCtccaAAtcctctccag | aaaaAaaAAGATATAGGAAaAAATAaaTCaatgAAgaaatgagct
121_511     : cAgtgctgtCAAAAACaGCgGAGGGacatGaactcatCatTTttt | aAaaaaaaaCAAAAACgGCaGAGGGtgtgGtggaaggCccTTgga
```

Alu, full-length, 51 sequences

```
Name         : Sequence around 5' junction                  | Sequence around 3' junction
3_180405     : tttctctAcctAAtAAAAAtGTTACggcCgggcgCggtggCtcac | aaaaaaaAaaaAAaAAAAAaGTTACaatCtatttCatattCattt
4_8108       : ttAAgcctttcAAAAGGTGgGCTCCgGccGGgcgcgGtGgCTcAc | aaAAaaaaaaaAAAAGGTGcGCTCCaGatGGattacGgGcCTaAa
4_124277     : AccAcAttAtttAagatAAaTGTTCggccgGgcgCggtggctcac | AaaAaAaaAaaaAgataAAtTGTTCaaaaGcaaCttcacagggt
4_176766     : tggctccctAAtAAAAGTAcAGTAAggccGGgcgcGgTggctCAC | aaaaaaaaaAAaAAAAGTAtAGTAAttagGGaataGcTattgCAC
4_187611     : ggtAAAgttTAAAACTTCAaTGTCCggcCggGcGcggTgGctcac | taaAAAaaaTAAAACTTCAgTGTCCttgCttGtGaaaTaGtgagg
5_200227     : gtctAttgcAgttAAAAAAtATTTGggccggGcgcggtgGctcac | aaaaAaaaaAaaaAAAAAAaATTTGatggttGgataaatGgatga
5_214126     : tttgcttAttgttAtagAAaTTTTTtggccGggcgcggTggcTcA | aaaaaaaAaaaaaAagaAAtTTTTTactggGtatttatTtatTtA
6_130582     : AgAAAagAAAgcTcAgtcAaAAAATtAAATGCGggccGgGCGcgG | AaAAAtaAAAaaTaAaaaAtAAAATaAAATGCGccggGcGCGatG
7_41848      : tActgAAtActttttttcttAAGAAggggccggGcgcggtggctc | aAaaaAAaAaaaaaaaaaaaAAGAAacatttctGattttcttaaa
7_63371      : AttAtAAcAttAAAaaACcaCTCCTggccgggcgcggtGgCtcac | AaaAaAAaAgaAAAccACtcCTCCTtaattcttatataGtCaaca
7_155113     : AttttgtcttttAAAAAAtTTTTCggccgggcgcggtggctCac | AacaaacaaacaaAAAAAAaTTTTCatttccaaataaacaacCtt
9_148010     : cAaAAttgtctTcttaAAAaAAATAggccgggcgcgGtgGctCac | aAtAAaataaaTaaatAAAtAAATAaagttactcaaGgtGggCtg
13_90964     : cAttAAActActttaaAATaAATTAggcCgggcgcgAtgGctCac | aAaaAAAaaAaaaattAATtAATTAattCaaaactcActGtaCtg
14_93366     : gAAtActAttAAAAAATTAaATGTGgCcgGgCgcgGtggctcAcg | aAAaAaaAaaAAAAAATTAcATGTGaCaaGaCataGgataatAat
16_56508     : AAggctAgttAtttAAAAAcATCTTggCcgggcgcggTGgcTCac | AAaaaaAaaaAaaaAAAAAaATCTTaaCaacaaaaatTGagTCtg
16_59524     : tAAtAAAtctgccttAttttAAAAAcTTGCgGCcggGcgcggtgg | cAAaAAAaaaaaaaaAaaaaAAAAAaTTGCtGCataGtattcaat
26_50390     : AttttcttctcAAtAAAAAtGAGAAggccgggcgTggtgGctcAc | AaaaaaaaaaaAAaAAAAAaGAGAAaatacctgcTcacaGtctAg
720_37       : tagaAaACcCtAgCTCcctcAAAACtTCTGAAATTggCCaggTGc | ggcgAgACtCcAtCTCaaaaAAAACcTCTGAAATTctCCccaTGg
3564_161     : tCaatTtccttAgttAAttcAAAAGcTCCCggccaggcgcagtgg | cCgtcTcaaaaAaaaAAaaaAAAAGtTCCCaatgcatgaatcaat
3896_39      : ttaAAAcAAAAtAAAGAAGtATGTTggccTggTgcggTgGctCac | aagAAAaAAAAaAAAGAAGcATGTTtattTcaTcattTtGtaCtt
9813_39      : cAAgAtggAAAAGAATAACaTTATAggcCaggcacggTggctcac | aAAaAaaaAAAAGAATAACgTTATAaaaCcaagtatcTctgcatg
10968_73     : caCggtAtgctcctAcAtctAAAGAtActaTtcTCTggcAgggtg | atCtcaAaaaaaaaAaAaaaAAAGAgAtacTatTCTccaAcataa
12413_27     : gctCCtActgtAAAACCTGtGAGGCgggaccaggcGtggTggTtc | aaaCCaAaaaaAAAACCTGgGAGGCaatttactagGaatTtcTgg
111348_14    : agAAcAAgAAcctgcAttttAAAAGcGagtcagctgaggccgggc | caAAaAAaAAaaaaaAcaaaAAAAGtGgagtcagctgaaatacat
117730_2     : agAtAAAAtggcttAAAaAtTTTAAgGCAGgcCGGgcAcGgTggc | tcAaAAAAaaaaaaAAAcAaTTTAAtGCAGttCGGaaAaGaTttt
122129_6     : AgctttcAtttAAAGTcTCtTTCAGgcTaggcgcggtggctCacg | AaaaaaaAagaAAAGTaTCcTTCAGctTgacaaaataaaaaCttt
195083_1     : ctttAAgatAttgtAGACAtTCTAGgCcgggcgcAgTgGctcacA | aacaAAacaAaacaAGACAcTCTAGtCaatcataAaTtGtaacaA
206052_9     : aattaactaaGAAAcCTGAaTGGAGgccaGgcACagtgacTcAca | ggaaggggtgGAAAgCTGAgTGGAGatagGaaACttactaTaAgt
357826_1     : tActtattTtgaAAatAagtTAGTTggccgggcgCggtggcTcac | aAtaataaTaatAAtaAgtaTAGTTaattctataCttacttTgga
11_29854     : AggAgActcAgttAAAACCaCACCCggccGGgCAcGgtgGcTcaC | AaaAaAaaaAaaaAAAACCgCACCCaagaGGtCAgGcgaGgTggC
14_33427     : ccAggctAcAtAAgAAAgAcAGGAGgccgggcgcggtGgctcAcG | aaAaaaaAaAaAAaAAAAaAaAGGAGatgtttgagacaGaggaAaG
15_58204     : agTtTgTAacacTtaAaAaaTATATggcCgggcgcggtggctcac | taTaTaTAtacaTatAtAtgTATATataCattgtactgatgggtg
24_26818     : cCCGgtTtAcAtAAtttAAcAAAAAtATTTggccgggCgcggTgg | tCCGtcTcAaAaAAaaaAAaAAAAAaATTTacagcaaCcattTat
2_224718     : ctgcttcAttAAAAAGAAGcAGATTggccgggcgCggtggctcAc | aaaaaaaAaaAAAAAGAAGaAGATTatgttcaacCatctctggAa
2_250216     : AAAttAgtttcAAtAAAAAtATTTGggccgggcgcggtggctcAC | AAAaaAaaaaaAAaAAAAAaATTTGaaaaaatattctgcctatAC
4_58189      : AagatatgActaAaAcAaAaATATAggccgGGcgcGGtggctcaC | AtatatatAtatAtAtAtAtATATAataaaGGaaaGGaaatgacC
5_23541      : tAttttcttAttctAtAAAaAGACAggctgggcgcgGTggCtcaC | aAaaaaaaaAaaaaAaAAAgAGACAaaagatataaaGTaaCaacC
5_47607      : TaAccAAtAAttcttAgctcAAAGAcATCAGTGACggccgggcgc | TcAaaAAaAAaaaaaAaaaaAAAGAaATCAGTGACtattactatt
9_171027     : AaAgcTtttTAAAAATGTGgAAATCgggctgggcAtggTggctca | AtAaaTaaaTAAAAATGTGcAAATCtcaaaaacaAaaaTaatatt
10_62494     : TgaagctctgAtgttgtctcAGAAAcGgAgGagGccgggcGcggt | TctcaaaaaaAaaaaaagaaAGAAAaGcAcGgaGgaacatGgtag
12_120882    : TAacaATAataAaAaATATaGTTGAggCcgggCgcGgtggctCAc | TAtatATAtatAtAtATATgGTTGAaaCttttCttGaattaaCAg
14_30541     : AgAAggAAtcAgAtAAAAAtGTTTGgCcgggcgcgGtggctcAcg | AaAAaaAAaaAaAaAAAAAaGTTTGaCtacaaagaGgataaaAga
19_78474     : tgttttcAgtAtAAAAAtAtTTTCTggccgggcgcGgtggCtCAC | aaaaaaaAaaAaAAAAAaAaTTTCTacatattttGtctcCcCAC
19_86389     : AggAAtActcAAGAAAGGCaTGCAGgccgggcgcggtggctcAcg | AaaAAaAaaaAAGAAAGGCtTGCAGatgtatatgttcacggtAat
20_67608     : atActttAttttAtAAtAAtGTGTAggccaGgcgcggTgGcTcAc | caAaaaaAaaaaAaAAaAAaGTGTAtatttGctcactTaGaTgAa
109_109      : AtCgAAAgcCAttAactAgaAAAAAtCCcTTtgaggccTGgcgcg | AaCaAAAaaCAaaAcaaAacAAAAAaCCtTTgagcagtTGctttt
809_115      : ggAaAgcatgtttAACAATtTCTAGgCcgggcgcGgtggcTcacg | aaAcAaacaaacaAACAATcTCTAGcCatctttaGtcctaTttta
8134_566     : cccAtggttTAAATtGGTatTTGTGgctgggcAcagtagctCATg | ataAataaaTAAATaGGTggTTGTGtggttatAtttggaggCATa
79017_2      : ctttAgtctgtAgCAtgttcAAAATtAtATAcATGgccgggcGcG | aaaaAaaaaacAaCAaaaaaAAAATgAgATAtATGtgaccagGaG
131937_13    : agaacAAgAActtgcattttAAAAGcGagtcagctgaggccgggc | ctccaAAaAAaaaaacaaaaAAAAGtGgagtcagctgaaatacat
219398_2     : AgAtAAAAtggcttAAAaAtTTTAAgGCAGgcCgGgcgcGgTggc | AaAaAAAAaaaaaaAAAcAaTTTAAtGCAGttCaGaaaaGaTttt
```

Alu, 5'-truncated, 18 sequences

```
Name         : Sequence around 5' junction                  | Sequence around 3' junction
4_92037      : ctgtgAggttcAtAAtttttAAGAAggccGagaCgggcggAtCAc | aaaaaAaaaaaAaAAaaaaaAAGAAtttaGgccCcttttttAgCAg
6_190485     : AAtAcAtgttAtAAAAATTaGCATAgcaggagaatggcGTgaAcc | AAaAaAaaaaAaAAAAATTtGCATAattcctttgacatGTtcAgt
10_87354     : atTtAgggAActttAAAAAtAAGTCggAgatcgAgaccatcctgg | tcTcAaaaAAaaaaAAAAAaAAGTCatAacattAtcaataagcaa
12_17927     : gctttAAAtAAAAATGTCTaAGAGTccggGcgCggTggctcaCgc | aaaaaAAAaAAAAATGTCTgAGAGTaataGgcCtaTttcttCta
19_60490     : AActAAtgtgAtttAAAACgCTTTCgaGcttGcAgtgAGccgAGa | AAaaAAaaaaAaaaAAAACaCTTTCatGgagGtAcaaAGataAGt
21_52817     : gctttAgaAtcatTctctctTTTAAgaTTgcGccAcTgCAGTccG | aaaaaAagAatcaTtctctcTTTAAtcTTcaGaaAaTcCAGTatG
3_198106     : tattttaatatTaaacAtAtAAAGAgACTACATTTCagccGgGcg | gcgagactccgTctcaAaAaAAAGAaACTACATTTCtaaaGtGaa
4_105209     : ttAttttAttAAAAAGTCCaCTCCAgccggGcgcGgTggctcacg | aaAaaaaAaaAAAAAGTCCgCTCCAagtatGtatGtTttgagtgt
6_112469     : AAgcAtctcAtTAATAATCaTATCAcTcacgcctgTaaTcccagC | AAaaAaaaaAaTAATAATCcTATCAgTgtgtttgtTcgTtagttC
9_134262     : gtgActcttGAAATCCTAAgTCCCAgtcCcagcTactcgggaggc | aaaAaaaaaGAAATCCTAAcTCCCAtaaCtttgTtgatttttcat
2_135839     : cAttcAAAAtAggAtAAAAcAATCTttAgccgggcgTgATgGcgG | aAaaaAAAAaAaaAaAAAAaAATCTaaAatttacttTaATaGttG
10_76266     : aTttAgttggtAttAcAtttAAAAGcTACTcgggagGCtgAggca | tTcaAaaaaaaAaaAaAaaaAAAAGtTACTgattctGCctAcaac
14_39379     : AgtcAgggctAgAAggttttAAGAGctaCtcgGGaGgctgagGca | AaaaAaaaaaAaAAaaaaaaAAGAGtggCagtGGgGaagtgaGgg
14_96019     : CtGagcattgAtAtgtgAAtAAAAAaggcgggcGgAtcAcgAggT | CcGtctcaaaAaAaaaaAAaAAAAAgtttctttGaAagAgcAttT
22_82029     : gCggttttAtAAcAgActgtGAAAGgCGGTTCACACCgaacCCgg | tCaaaaaaAaAAaAaAaaaaGAAAGaCGGTTCACACCttcaCCct
```

```
26_52213   : ctTttactgCtAAggcAtttAAAAGgcTcAcgcctgtAatCccag | acTccgtctCaAAaaaAaaaAAAAGaaTaAtttactcAcaCtgct
19328_40   : ctgcAtttAAAATTATAAGtGCTCAgccAgGcaTggtggcTcacg | aaaaAaaaAAAATTATAAGaGCTCAatgAaGtgTaaatatTtgtt
1145855_1  : AActtctAtgAagcatcAttAGAATgTcTtTaaAtggcTCAcGCc | AAaaatgAatAgaagaaAaaAGAATaTgTcTttAaaatTCAaGCa
```

Additional file 3. Sequences around 5-bp assumed TSDs. Nucleotides identical between 5' and 3' junctions are in uppercase. TSDs if we allow a 1-bp nucleotide substitution sandwiched by ≥2-bp stretches of homologous nucleotides are shaded in yellow.
