# Supplementary material for: Different integration site structures between L1 protein-mediated retrotransposition in cis and retrotransposition in trans
Source: Mob DNA. 2010 Jul 8;1:17. doi: 10.1186/1759-8753-1-17 (PMC2912911; doi:10.1186/1759-8753-1-17)
Supplement: Additional file 4 — Sequences around 6-bp assumed target site duplications. [file 1759-8753-1-17-S4.PDF]

L1, full-length, 4 sequences.

Name	Sequence around 5' junction	Sequence around 3' junction
7_51123	tttcAAAAgaagAcAAcAttAAAAATaAAAGAACAGggaGgAgcca	aaaaAAAAattaAaAAaAaaAAAAATgAAAGAACAGatgGaAtaag
2_222865	acAtctccAttcAttAAAGtTTTTGGggggggaggagCcaagaTGg	ttAaaaaaAaaaAaaAAAGcTTTTGGctattctctgtCtcctcTGc
2_116086	tcCttgccAgcAtctcActtAAAGAAgGTACCATGACAGagCcaag	aaCactgaAaaAaaaaAaaaAAAGAAtGTACCATGACAGccCtcta
7_157327	gtcctacctttAAAATTTTaAAATGCgcAAgaTgGcCgaaTAgGAa	aattggaaaaaAAAATTTTtAAATGCttAAagTtGtCtgtTAtGAt

L1, 5’-truncated, 64 sequences.

Name	Sequence around 5' junction	Sequence around 3' junction
11_60222	AtagTggatgAgtAcagtAtAAAAAAaAaaAAaaCacATgaAgaaa	AgtaTaataaAaaAattaAaAAAAAAtAtcAAggCtaATtgAactg
7_66250	catAgAtAActggGcttttttAAAATGaaAaaATgCTcatcaTcact	atcAaAaAAaaaaGaaaaaaAAAATGccAgcATtCTgtgttTtcaa
2_33251	tgactctcAAAAAaatctttAAAAATaaaTCaTgctgCtaTaaaga	aataaaatAAAAAttaaaaaAAAAATcctTCtTatctCagTctgag
3_76574	tgcAttgggccAAgtttttttTAAAAAaCCATTGCtTTttaaAacca	aaaAaaaaaagAAagaaaaaTAAAAAgCCATTGCaTTagtcAgagc
9_143883	TAttAgAAgcAAttcctAgtAAGAAAaAccaAAcAccgcaTaTtct	TAaaAaAAaaAAaaaaaAaaAAGAAAgAgttAAtAtaatgTgTcag
8_68631	tCTaAcAAAAtAAgActtttAAAAAAaacCatcattCtCaGtAaaC	gCTtAaAAAAaAAaAaaaaaAAAAAAgtaCccacaaCaCtGgAgcC
9_32442	cttAaAtTccggttAAAaaaTTTTTCaagGatcAacaAaATtgata	agaAgAcTttaaaaAAAtttTTTTTCtttGtggAtatAcATatgac
20_58202	AtaAgaAcatgaAtAttAAgAATACTaatTaAactAaaGaGcttct	AatAatAataatAaAaaAAaAATACTtcgTtAgtcAggGtGtggtg
1_140943	GttggtatAgttctttAAAgAATTTCaattttCaaCccagaatttc	GaattaggAagggaaaAAAaAATTTCttcccaCtgCtgctgtagga
5_114619	AtAAcAtAcAAttAtgggAtAAAAGCacAtgtacccTaaAAcTtag	AaAAaAaAaAAaaAaaaaAaAAAAGCttAatgcatgTgtAAtTgga
15_48976	tggcttcAgAAtgtttcAttAAGAAAactaGAaataccaTttgacc	aaaggggAaAAaaaaaaAaaAAGAAAggagGAttctgttTggatag
15_97364	agttcAAgAcccAgcctAttAACAATaCtaGaTgacgagTtagtgg	tcaaaAAaAaaaAaaaaAaaAACAATgCagGcTcttagtTggaacc
1_88850	cccacAcAAgccAgAtttttAAGAAAactaTcgcaagaaCaaaaAa	atgttAaAAaaaAaAaaaaaAAGAAAgtctTgctccttcCcttcAc
592_132	AcTTatttgGtgtctttAAgAACAAAaGTGggcgaagGAcatgaAc	AtTTcaaaaGgaaaaaaAAaAACAAAtGTGaaaatgcGAttgatAa
1_228457	AAtgcTAAgggtccctgAatGAAACAataTTcaAcAttcttaAaga	AAaaaTAAaaaaaaaaaAgaGAAACAggcTTttAtAaataagAgac
1_244834	ActtAgAacttctgtAtgttGAAAAAatgActAtAaAtcAtgctGc	AaaaAcAttaaaaaaAaaaaGAAAAAtaaAtaAcAcAgtAgataGa
8_77461	aTttAaTtcAgTgAAAAAAtATTCAAaTgtggcAcAtatacAccaT	tTaaAgTatAaTaAAAAAAaATTCAAgTactatAaAatataAatgT
10_104211	TtTttactttAAccAAcgAtGAAACTatTccaaaactgAccaCAta	TaTaataaaaAAaaAAaaAaGAAACTcaTtacttttaaAaggCAag
4_178671	ctggtAaTtgAAgAtgTtgtAAAATAcaTAcgtaactaacctgcAt	taaaaAgTaaAAaAaaTaaaAAAATAtgTAatatttaggagaatAa
6_15203	ccaggTgAAAAgggAAgttaAGAGAAccaAcGcaAAtgtccaacaa	tataaTaAAAAaaaAAaaagAGAGAAtttAgGggAAacaaatgtgc
7_109264	gcaAAcAtAtttAgaggtttTAAAAAcTgAAcaAccTgcTcctGaA	aatAAaAaAaaaAataaaaaTAAAAAtTaAAttAgtTcaTttgGgA
13_81546	aggtAAAgtAtActgtGAggAAGAAAgaAGACatttATgCAgccaA	taaaAAAaaAaAaaaaGAaaAAGAAAtcAGACccaaATcCAaaatA
7_99350	AtAtgATattAtctcAAtttAAAAAAgAgcttctgCacagcaaaag	AaAacATtaaAaaaaAAaaaAAAAAAaAcacaacaCtgcttctttt
68271_7	atTccatAgAgAttTtAAgaCCCATAgcaaggaCttcatgacaAac	tgTttcaAaAaAaaTaAAacCCCATAaatctccCaaagcagttAct
2_140275	ttAAAggcTcAAtcccgtttAAAACTgCAcatgtAcccTaAAactt	aaAAAaaaTaAAataaaaaaAAAACTtCAacctcAaatTcAAtgca
11_119562	AAAgcAAAgAtttgctAtttAGAAGTggAcacaGgAAGgggaatAt	AAAaaAAAaAaaaaaaAaaaAGAAGTtaAggttGcAAGatcttaAg
6_20616	gccatgcaaaaCaaCtcAtcAAAAGTggggagggaggaggggggAg	atatgtatcccCccCaaAaaAAAAGTatatcccatactttatttAt
14_103754	AgcattAatgAAAtGtgAAtAAGAAAgtgggggaggggGgagggA	AaataaAtaaAAAgGaaAAaAAGAAAacatttcttaactGctttaA
2_215127	ttgtAttttgtctttAAAAaCACTGTggTggtggggTcggggGagG	aaaaAaacaaaaaaaAAAAcCACTGTctTaaatactTaaaaaGgtG
13_49396	AAcctAAAAggtAgttcAttAAAAATtacaaAgTcaGgAaAcaacA	AAtaaAAAAaaaAaagaAaaAAAAATctgccAtTttGcAgAgcctA
4004_205	tActcTgtattAGAAATAAcAGATTAttCtTtgacAaaccTgAaAa	aAtaaTaataaAGAAATAAtAGATTAcaCaTcttgAtcaaTtAtAt
5_29804	ggTctccttAgAAcccttAcAAAGAAtgaaTccaggAgctggtttt	ttTaaaaaaAaAAaaaaaAgAAAGAAatttTtgcaaAcaaacacaa
14_122522	gggAtgtgAatAtatcCcAtAGAAACtgGgaTGtAtttcaaaAtAa	aaaAaaaaAgaAatatCaAaAGAAACcaGagTGcAggcttttAaAc
20_49112	taatgggggcAccAAAtgAaATATTTaaaCTatcgcaagaaCaaaa	agtataatttAaaAAAatAtATATTTctcCTggaaagttccCtggg
21_21585	cAggAcctccttccAAggttAAGAAGaCatttATgcagCcaAAaaa	aAaaAaaaaaaaaaAAaaaaAAGAAGcCgcaaATatcaCttAAttt
9_49238	AGccatcagAAtAAcAgtAgAAGCGTtAccAaAcaccgcATATtcT	AGtataattAAaAAaAaaAaAAGCGTcAagAcAgttactATATgaT
10_76343	TTcAAgAttttctAAtAtttAGAAATagcatggcAcATgtATacaT	TTaAAaAaagaaaAAaAaaaAGAAATttgccaatAtATaaATgacT
40403_3	gaAtAttttctcctgtttTcTAGATTagTGgggTggggggAGgggg	atAaAaaaataaaaagaaTaTAGATTtaTGcttTacatttAGataa
7_7871	aAgctcAcccccttcttttAAATGTaTacataTgtaacTaaCCtg	tAaagtAtaattaaaaaaaaAAATGTtTcacgcTaggttTgcCCaa
11_83709	ccctttgttcctattAtttcAAGAATatgTaActAacctgcAcaaT	taaaaataaaataaAaaaaAAGAATgctTgAacAgtgaagAgtcT
10_39186	TctAaAAAATacctgtctttAAGATTatgTAactaacctgcacaAt	TaaAtAAAATtaaaaaaaaaAAGATTcaaTAgtatttaattttcAa
4_54900	AcAaccacAcTttAAtctctAAAACTcataggtgggaattgaacaa	AaAgtacaAtTaaAAaaaaaAAAACTttccaaataattcattttt
3564_118	ataAAagaatAtAAAGATGtGTGCATcATgTaTacataTgcAacaA	tatAAttttaAaAAAGATGcGTGCATtATaTtTtatgtTttAttcA
2_2133	attAtgAAAccAtAcctcAtAGAAAAccaaacAccgcATatTctca	taaAaaAAAaaAaAaaaaAaAGAAAAttgtcaAggaaATtcTtgat
2_240264	gAggataAttgaAAtcttAgAAGAAAcCataAaaaccCTAgAagaA	aAttcggAaaagAAaaaaAaAAGAAAgCtagAgggagCTAaActtA
4_80261	ccAgAAAgtAtccctcttttAAAACAcCgcATattcTcactcatag	gaAaAAAaaAaaaaaaaaaaAAAACAaCtaATtaaaTtttattgta
8_47549	TtgcATgtcttggagAtAttAAAACTcCggggActgttGtgGggtg	TataATaataaaagaAaAaaAAAACTaCtttaAacaaaGatGtcct
16_68250	cacAAAgctAAattgAatttAAAATGctAgaTGAtgAgttaGtGgg	ttaAAAaaaAAgaaaAgaaaAAAATGaaAacTGAaaAaaagGgGct
2_147390	cAAgAttctAgggtcttAgtAGCAAActcaTctGacAaAGGGctaa	tAAaAaataAaaaaaaAaaAGCAAAgctcTgaGgaAgAGGGagtg
3_87781	tcAtcAgAgtggAtctgtttGAAAAAaTtctgTgGtgGggaggggg	caAaaAaAaaaaAgaaaaaaGAAAAAtTctccTaGcaGtttaatta
14_27572	gcctcaTccATtacagAAgcAAAAGAcTtACaagggATgtgaagga	ttaaagTatATatataAAaaAAAAGAgTgACtgaacATtgcttttt
5_46714	AagtcActtcccggAAggAgAAAAGGgAatatcaTActcTggGgaC	AgcaaAaaaaaaaaAAaaAaAAAAGGaAggcatcTAtagTatGatC
1_97594	AgtttcAAattttctAActtAAAAATgagAtcAcatgGacAcAGGa	AaaaaaAAtcaaaaaAAaaaAAAAATtgaAatAggctGggAgAGGt
11_75674	gtTccaaagggAgAAAttttAAAAAAcAGGAAGAAGAtgaaACTgg	taTaatttaaaAaAAAaaaaAAAAAAgAGGAAGAAGAaaggACTaa
5_215388	tgTagAtcAttttggActtAAAATGggagaTatAtctCatgCtAg	aaTtaAaaAaaaaaaaAaaaAAAATGaagctTtaActgCcccCaAa
7_16192	tttAAaAAcAAaAttgcAAtGAATATggcAaTcattaaaaAgtcag	aaaAAtAAaAAtAaaaaAAaGAATATcctAgTgcacttttAagatt
12_23206	gcTaAttcctAttcctccttAGAAATggccAacAgagaaATgcaAa	aaTtAaaaaaAaaaaaaaaaAGAAATttgtAtaAcctcgATaacAc
14_14540	AtgtcAActctgAtgTttAgAAATAAgggggAgggggggagggatag	AaaaaAAaaaaaAaaTaaAtAAATAAatatcAacaatatattgata
14_121906	cTAattTttgAttggtttatAAATTGgtgggaaTtgAaCaaTgAta	gTAtaaTaaaAaaaaaagtaAAATTGtactattTgcAcCtcTtAct
10_141730	AActtcAtTAggtgAAAgAcAATATCtAgGaaTCcaActtacaaGg	AAaaatAaTAataaAAAaAaAATATCaAtGttTCttAaagtgtgGt
14_55784	AAtaTgttgtTcggActAAtAAAAATtCTcActCAtaggtgggAAT	AAacTtaaagTataAtaAAaAAAAATaCTaAtcCAatatacttAAT
7_114548	TtcTgtgcttAttAtccAgtAAAGAAtgagatCAcAtGgAcacaGg	TaaTaaaaagAaaAaaaAaaAAAGAAatgacaCAgAaGaAatttGa

```
2_85998      : gggAgAggAAagcattttgcAAAATAtttaCaAgaAaAAacAAaAc | taaAaAaaAAccttgaaaaaAAAATAccagCtAtgAgAAtaAAcAa
6_32914      : aAtcttggAggtcttggtAtAAAAGAtTtgaCccaGCcatCccAtt | tAataaaaAaaaagaaaaAaAAAAGAcTaacCaggGCtgcCaaAaa
```

L1, 5’-inverted, 6 sequences

```
Name         : Sequence around 5' junction                   | Sequence around 3' junction
3_124262     : ggagttgAggcAAccAcAgcAAGAAAaGCAGGAACATTCTTTTgtA | cttaaaaAaaaAAaaAaAaaAAGAAAgGCAGGAACATTCTTTTaaA
4_29103      : gtgccttAAGAACACCTCAgTTTGTTcTgTtccaTTgATcTatatc | aaaaaaaAAGAACACCTCAaTTTGTTaTtTcaagTTcATtTtctat
16_57771     : tgtcAttAAtAAAgttAAAgAGAGTCgaggaatgtatccATtTctt | aaaaAaaAAaAAAaaaAAAaAGAGTCttcccccaggggaATaTgcc
13_103916    : gtcAAtgAAgAtcAgcctttAAAAAAcATAGGCTCTGttTTggTtg | aagAAaaAAaAgaAaaagaaAAAAAAaATAGGCTCTGgaTTcaTga
1_195928     : AgtAgAtgcAtAAGAACATtGAGGCCtgAATggtAaTgCCtaGgtt | AaaAaAaaaAaAAGAACATcGAGGCCaaAATtacAgTcCCagGagc
4_120595     : tTttagccAttcttAtAAAcATTCCAccAacagTgTaaaagTGttc | aTaattaaAaaaaaAaAAAaATTCCAttAgtttTcTtctccTGaaa
```

Alu, full-length, 69 sequences

```
Name         : Sequence around 5' junction                   | Sequence around 3' junction
1_173119     : AcccсttcctcccAgcAttcAGAACAcGCAGTAAGgccgggcgCgg | AaaaaaaaaaaaAaaAaaaAGAACAgGCAGTAAGagtcttacCat
1_214548     : AtgtAtAtAtgtttAAAGAaATGTGTggggccgggcgcggTggctC | AaaaAaAaAaaaaaAAAGAcATGTGTacattacatgtatcTattaC
2_99372      : AgAtcAAgcccAgAAAttAtAAGAAGtTTACAGAAGgccggGCGcg | AaAaaAAaaaaAaAAAaaAaAGAAGgTTACAGAAGatattGCGtt
2_114454     : AtAAgttAAtttAAAAAgAtGACGAAggccgggcgCggTggcTcaT | AaAAaaaAAaaaAAAAAaAaGACGAAaataatataCaaTtttTtcT
3_86113      : ttggAgActAAAActAttttAAAAAAatgCcggcCgGgcgcgGtGg | aaaaAaAaaAAAAaaAaaaaAAAAAAtgcCttaaCtGtatttGaGt
3_127627     : tctgttctttttAAAAgAGaTTAATAgGccgGgcgcGgTggctcac | aaaaaaaaaaaaAAAAaAGgTTAATAtGtaaGattaGcTtttgtta
3_141169     : AAcgAtgggtAAGAATAAGcCATCTGggccgGgcGcggTGgctCaC | AAaaAaaaaaAAGAATAAGgCATCTGaatgaGaaGaacTGtgaCtC
5_14952      : tccttgtgttAtcatAAAAcCGTAACgGccGggcgcggTgGcTCAc | aaaaaaaaaaAaacaAAAAaCGTAACaGttGacatgctTtGtTCAa
7_78637      : AgAAttgcAcAgAgAAGATtTAACAAggccggGcgCggtggctcac | AaAAaaaaAaAaAaAAGATaTAACAAatggatGaaCcaccttgtgt
8_79834      : tgcAtgCttttTggtgAAAtAAAAAAtagtcggccGggcgcgGTGG | gagActCcgccTcaaaAAAaAAAAAAatagtcatgGcaacgtGTGG
8_124574     : AAAAAttAgattAAAaagAaAAAGGAggccGgGcgcggtgGCTcAc | AAAAAaaAagaaAAAcgaAgAAAGGAattgGaGgatatctGCTgAt
8_127829     : AgtAAAtcAgAgccAtgcAtGAAAAGtCCCCAGACggccGggCgcg | AaaAAAaaAaAaaaAaaaAaGAAAAGcCCCCAGACttatGcaCagc
9_100204     : tgAgtcttAtcAAAAgAAgtGAGAGGggccgggcgcggtggCtcac | aaAaaaaaAaaAAAAaAAagGAGAGGatagatataattaaaCatct
10_84550     : CttTtgaAAAttgAtttttttAAAAAGtCCTTTACAggccgggcgca | CcgTctcAAAaaaAaaaaaaAAAAAGgCCTTTACAattttttatttt
11_98192     : tgtAtAgctcttAAATCAGgCACTGGccgggcGcGgTggcTcAcgc | aaaAaAaaaaaaAAATCAGaCACTGGtgcaaaGtGcTataTtAaca
11_114362    : tAAtttctttAAAAAAgAtAACTTCggccaggcgcggtggcTcaC | aAAaaaaaaaaAAAAAAaAaAACTTCactttttacttaaatgTtcC
12_58389     : ctttcAtAAgttAAAAATTtGGGGCTgGgcGcggtgGCtCAcgtct | aaaaaAaAAaaaAAAAATTgGGGGCTtGaaGttaaaGCaCAttgtc
12_67824     : AtccAgttAAcAgtttcAAtATCATCggccggGcgcGgTggCtcaC | AaaaAaaaAAaAaaaaaAAaATCATCtatataGataGaTccCaagC
13_33819     : tgcAttAttgtccctctAttAAAAAAtgcTtggccgGgCgcGgtgg | aaaAaaAaaaaaaaaaaAaaAAAAAAgctTatttgtGaCttGtaca
13_51814     : ctgtgttttccttctAcAttAAGAATtTTCATTCTGggccgggCgc | aaaaaaaaaaaaaaaAaAaaAAGAATcTTCATTCTGaaggacaCag
13_78651     : AgAAtggAttAGAAAAATAcATATCTggccGggcGcGgTggctcAC | AaAAaaaAaaAGAAAAATAtATATCTatagGtctGtGcTataggAC
13_131390    : AAtAggtgtAcAtgtGAAAtTATTTTggccgGGcgCgGtggctcac | AAaAaaaaaAaAaaaGAAAaTATTTTaaagtGGttCaGgaaaattg
14_10457     : gtAttcAgtAtttAAgAgAgCTTGCTggggcCgggcgcggtGGctc | aaAaaaAaaAaaaAAaAaAaCTTGCTttctgCtaattgaaaGGtaa
14_56503     : AggAAActAtAAAAAGTTTtTTTCTGgcCgggcgcgGtggctcacg | AaaAAAaaAaAAAAAGTTTcTTTCTGtaCctttattGgtttagttt
14_105495    : aaaAgtggcAAtAAAgAAAcAGAATGgGCcgggcgCggTggCTcac | ctcAaaaaaAAaAAAaAAAaAGAATGaGCaaaaacCttTtaCTact
17_30246     : gAtgAgAcAAccAtcAAAAtGGAAAAggccgggCgCgGtGGctcAc | aAaaAaAaAAaaAaaAAAAaGGAAAAaaaaatcCtCtGgGGaagAg
17_65054     : CAAAttctAAttAAAAAAAtACTTTAgGccGGgcgcggtggctcaC | CAAAaaaaAAaaAAAAAAAaACTTTAtGgaGGaattaaaaagatgC
17_70510     : gtccAttgtgAtAAAAAgAgCCTGTAgGccgggCgCggtGGctcaC | aaaaAaaaaaAaAAAAAaAaCCTGTAaGaaaccCtCatgGGtgacC
19_90402     : aaagTaggAgtttAAcAAAcATCTATaggccgggcgcGgtgGctca | cgtcTcaaAaaaaAAaAAAaATCTATgactgaattatGaatGaatg
19_103338    : aCccagaActcttcAgAAtcAAAAAGgCCAAAATAggccgggcGCg | cCgtctcAaaaaaaAaAAaaAAAAAGaCCAAAATAaatgttaaGCa
22_50249     : cAcAgActccAccAAcAAttAAGAACaGGTTCAGGGgccGGGcgcg | aAaAaAaaaaAaaAAaAAaaAAGAACgGGTTCAGGGcttGGGaaaa
1461_199     : tGgaTCctgAAttcgAgAAtGAAAAAtAAATTGGAggccgggCacg | cGtcTCaaaAAaaaaAaAAaGAAAAAcAAATTGGActaaattCtat
21873_3      : gccttgAAAAAAGTAGATAgAATAGGcTgggcGcGgtGgctcacgc | aaaaaaAAAAAAGTAGATAtAATAGGgTaaagGaGaaGtggtggta
27333_27     : cttttcttAAAAAaTATCAaTAATTTggccAggTgcggTggctcat | taaaaaaaAAAAAtTATCAgTAATTTaatgAtaTttctTaagaaga
43887_7      : tgcAgtgAgtAtttAtgAAaTCTTTAggccgggcAcggtggctCac | caaAaaaAaaAaaaAaaAAgTCTTTAaaatttgAaataaaggCgt
133438_2     : gtggAAAAgttAAAaTATTtAGTGGCggCcaAgaGcaGTggctcAc | cacaAAAAaaaAAAgTATTcAGTGGCacCtgAagGgtGTtcaggAt
160058_12    : AtcAAAtctagcAtttAAAgACTGGCacgGctggGcGcagTAgctc | AaaAAAaaacaaAaccAAAaACTGGCttaGtaaaGgGggaTActga
216051_3     : ttcAtttAAAttAaAgAAAaAAACCTggccgggcgcGgtGgctCAc | aaaAaaaAAAaaAcAaAAAtAAACCTtttaaataaaGtgGttaCAt
348748_1     : tgtATcAgggAcatggAAAgAAAAAAacaTTTTtAggcTgggcgtT | aaaATaAaaaAataaaAAAtAAAAAAcatTTTTaAaaaTacataaT
736032_1     : TaAAtcAAgAAActcAAAAcTCACAAaTAtAgggcTgggCgCgGTg | TcAAaaAAaAAAaaaAAAAtTCACAAgTAgAatctTcaaCtCtGTa
1_182684     : ggtAAAgcAtgAtcttAtgtAAGAAGtGTTTGGAAtGgccgggcgC | tcaAAAaaAaaAaaaaAaaaAAGAAGgGTTTGGAAaGaagatagcC
3_87255      : AtAgtgttttcAAAgActtTGTTCAggcCGggcGcggtggctCaC | AaAaaaaaaaaAAAaAgacTGTTCAtttCGcctGtccacttaCcC
6_99527      : AAtgtggtggtttAAgAAAtGGTTTGggccgggcgcGgtggCTcac | AAaaaaaaaaaaaAAaAAAaGGTTTGataattaaaaGagttCTttt
17_30666     : ctccAgctctttAAAACATaATGATGggccgGgcGcggtgGctcaC | aaaaAaaaaaaaAAAACATgATGATGatgatGatGataaaGtcatC
24_38354     : gAttAcAActaAAGAAGTTgTCTTTTggCcGgGcgtggTggCtCac | aAaaAaAAaagAAGAAGTTaTCTTTTctCaGtGttgctTctCcCta
32_5782      : cccctggcAttAAAAGCTGcAGGCAAggccggGCgCggtggctCac | aaaaaaaaAaaAAAAGCTGtAGGCAAaaggtaGCtCtactctcCtt
1381_436     : AtgggAAgcAgAAAtggAgtAAGAGGtgGACATGTggccagGtgtg | AaaaaAAaaAaAAAaaaAaaAAGAGGctGACATGTatgtgaGgaca
7368_229     : gcTcctctgAaAaaTATCCaAAGGGGtgGcagGGTAcggtggctcA | taTatatatAtAtgTATCCcAAGGGGcaGggaGGTAtaccataatA
13699_31     : cccAAAtgctcatAGAAcatAAATACtggacCagGtgTggtgGctc | aaaAAAaaaaagaAGAAacaAAATACccactCctGaaTcacaGaca
1_25244      : CAgtAtgAttcAAcAcAcAtAAAAAGcCAtTTGGggccGGgcgcgg | CAaaAaaAaaaAAaAaAaAaAAAAAGaCAaTTGGtattGGcaattt
1_276165     : AgAcAtttggtAtAAgaAtaCACATCggccgggcgcggtGgctcac | AaAaAaaaaaaAaAAagAatCACATCacagtactaattaGaaaagt
3_93052      : ttctaAgtgAAAtAAAAAAtAATACAggccggGcgcggtggcTcaC | aaaagAaaaAAAaAAAAAAaAATACAaaatttGgttaaatatTtgC
3_96131      : TgTgAAtctAtAttAAAAAtTTTAGGcCgggcgccgtgGcgcacAc | TcTcAAaaaAaAaaAAAAAaTTTAGGgCaaaaattaaaGgaagtAa
3_183360     : cAgctAAAAAAAAGTCAACaATTAGAggccgggcgCggTggctcac | aAaaaAAAAAAAAGTCAACgATTAGAatgtaaattCatTccaatta
5_149969     : ttcttcctttt AAAAAAAAtCTTTAGgccggGcGcggtgGctcacG | aaaaaaaaaaaAAAAAAAAaCTTTAGtaaaaGtGtaacaGtcaggG
7_91255      : agCAtttgtAAAccAtAAAaCTTTCAgCTtggCCgGGCgCgGtggC | ctCAaaaaaAAAaaAaAAAcCTTTCAtCTctaCCaGGCtCaGaatC
10_145052    : GTgTggggAgttggtAgAAtAAAAAAtTaAAcacggccgggcgcgg | GTcTcaaaAaaaaaaAaAAaAAAAAAaTtAAacacaagaatgaaac
11_69436     : ttgtttttAAAAAAAcAAcAAAATTggccgggcgcGgtggctcAc | aaaaaaaaaAAAAAAAaAAaAAAATTaatgataaaaGaaactgaAa
```

```
15_33778    : AAAAAtgctgtAttAAAAAcATCTCCggCCgggcGcGGtGgctcAc | AAAAAaaaaaaAaaAAAAAaATCTCCaaCCaaagGaGGgGaaaaAa
26_44240    : tctgAggcctttAAAAAAAtGATCCTggccgggcgCggtgGctcAc | aaaaAaaaaaaaAAAAAAAaGATCCTttgatatatCttctGaaaAg
701_64      : gTtAtcActtAggAtAAtgaAAGAAGtGTGATTggcCgGGCgcggt | cTcAaaAaaaAaaAaAAaagAAGAAGcGTGATTaaaCaGGCaaatg
6126_114    : ggAAAttggAgtttAAAAGgTATATTatTGctgGctggGCatgGTg | aaAAAaaaaAaaaaAAAAGaTATATTgcTGaacGacaaGCcatGTt
6937_328    : ccAatgACcttaAaACACAgGGCAGGgccgggcgtggtggctCacG | aaAcacACacacAcACACAaGGCAGGtaaaaattatagatggCttG
42747_4     : tgcAgtgAgtAtttAtgAAaTCTTTAggccgggcAcggtggctCaT | aaaAaaaAaaAaaaAaaAAgTCTTTAaaattttgAaataaaggCgT
88675_65    : gtcAgcAcccAgtgtctcttAAAAGGcCCAGtgtCtcttcaAgGCt | aaaAaaAaaaAaaaaaaaaaAAAAGGaCCAGgacCagacagAaGCc
109208_17   : AtattgTtTtcAAAAAcAGaTGGTAGgCcAggtgtggtggCtcaca | ActccaTcTcaAAAAAaAGtTGGTAGaCaAaactataatcCagcat
19_85053    : AAAccctggctAAAGAAAAtAAATGGgccGggcgcGgTGgctcaCg | AAAaaaaaaaaAAAGAAAAgAAATGGtttGtatatGaTGctctgCt
109_4585    : aAccgccAcAgAaaAcaTccACTTTCggccagGtgTggtagTTCac | gAaatgaAgAcAtcAacTtaACTTTCacatttGccTccattTTCtt
35098_4     : attttacTtaaAAacatgctAAGGTCggCcAggcgtggtggctccc | caaaccaTgttAAggtctacAAGGTCtaCaActtagtcatttctta
```

Alu, 5'-truncated, 26 sequences

```
Name        : Sequence around 5' junction                    | Sequence around 3' junction
2_140322    : ttctttgtgttAttttAAAtTTTGTGccgggcgCgGtgGctcAcgc | aaaaaaaaaaaAaaaaAAAaTTTGTGtatatttCcGgtGgggAtta
2_243396    : ggggccctAAAgctAtcAttAGAAATtAACAGGGCAttgggagGcc | aaaaaaaaAAAaaaAaaAaaAGAAATgAACAGGGCAgaaactaGgg
3_220385    : tttccAAgAtAAAttAAAAtGAAACCccGcctctactAaaAAtaca | aaaaaAAaAaAAAaaAAAAaGAAACCaaGtaagggagAtcAAggag
4_65629     : AggAAAAcAtAAAAAtAAAtGAACTAgcAcTTtgggaggccgAGgc | AaaAAAAaAaAAAAAaAAAaGAACTAatAaTTcaattcaagaAGtt
5_5575      : ttggAAAtAtAAAAAGTACaGGAAAGatCccgccaCtgcactCCag | aaaaAAAaAaAAAAAGTACcGGAAAGcaCataattCcatgtcCCct
5_39427     : ttctcAtAtAtAAGAGCTAcTGCAGCcgggcGcggTggcTcaCgcc | aaaaaAaAaAaAAGAGCTAtTGCAGCaatatGttcTttgTagCcaa
5_161874    : ctAgttctAttAcAAAAtTgGGCAATgggaGgccgagAcgggCgGa | aaAaaaaaAaaAaAAAAaTtGGCAATatttGcaactcAatctCtGt
5_181685    : tgcAttAggctgggAtctttAAAAGAggccgagGCgggtggaTcat | aaaAaaAaaaaaaaAaaaaaAAAAGAtttgagtGCtaagcttTtgg
7_55110     : cAtgctcAgAgAAgAtggAtAAACAAttgggaGgccgaggCgGgcg | aAaaaaaAaAaAAaAaaaAaAAACAAgaccagGtgaatttCtGctt
9_46664     : ctccgAgccttAAAGAGTGgCTGATCcGgGagCggtGgcGGgctcc | aaaaaAaaaaaAAAGAGTGaCTGATCtGtGttCatgGatGGatgag
10_178741   : AgtgAgtAAgAAATAAATAcACTTTTtagtggcGggcgcctgtAGt | AaaaAtaAAtAAATAAATAaACTTTTattgttgGaagctgctgAGa
12_87353    : AtttttAAgtgtgAttgAtgAAAAAAcATGCCCAGACCTcgCggtg | AaaaaaAAaaaaaAaaaAaaAAAAAAaATGCCCAGACCTacCcaga
13_108617   : ggggtAcAAAAActgtctttAAAAGCcGTCtCtactAaaAatacaa | tctcaAaAAAAAaaaaaaaaAAAAGCtGTCaCatggAttAgacttg
22_80806    : AAAgctggAAAAAgAacagcAGATCAtGccgggcgcGGtggcTcaC | AAAcaaaaAAAAAaAcagcaAGATCAgGggcttgagGGcacaTggC
82_491      : AttatAAgcAtAAAtAAtttAAAAGTtTGAggCcagcactTtggga | AcctcAAaaAaAAAaAAaaaAAAAGTgTGAaaCatttgacTgtatg
4445_44     : gagaaAgtgGggtAtAAAAaAGCAGAaGATTgccaggcacggtGGc | ccatcAaaaGaaaAaAAAAcAGCAGAgGATTttactcatactgGGa
31996_105   : tgATgtAgcattAAAAAGAaTTTGTCagctgAgcaCAgTggctcAC | aaATaaAaacaaAAAAAGAgTTTGTCtagatAatgCAaTattagAC
191468_3    : gTtttcAgtgAAAAAAATGtTATCAGccgcacacggTgGctcacgC | cTcaaaAaaaAAAAAAATGaTATCAGtacttactcaTtGtgatgtC
8_39885     : AAAAAAAActgAAgcAgcttAAAAAAgGcggagcctgcagtGagcc | AAAAAAAAaaaAAaaAaaaaAAAAAAtGtatgtgggaagtgGctgg
8_16464     : gtAtgtAccAtAAGAAGTAcTTGGACcTtTGggaggccGaggcggg | aaAaaaAaaAaAAGAAGTAaTTGGACaTgTGaacacatGtacaata
28_47446    : CtTtgatAgAggtgAttttAAAAGAgTAGGAGAaaCacaGtgAaa | CgTctcaAaAaaaaAaaaaaAAAAGAaTAGGAGAggCtggGgcAgt
100_2752    : gccAaactatAtTAAAAATgATTCCAgccAGgcAcAgTggctCgcg | taaAcgaataAaTAAAAATaATTCCActtAGcaAaAaTaataCtta
4834_104    : gttgACtgttAAAttCaAAaCTAGTTgTAgcCgggcgggGtgGctc | acaaACaaaaAAAaaCcAAgCTAGTTcTAtaCttttacaGatGgaa
205855_4    : cccttcccAgAAtAgAAATcTCCTCTtGtccaggcaGtgTctcacG | aaaaaaagAaAAgAaAAATtTCCTCTcGcttctctgGccTtgtgaG
4156_127    : AAActcAgcAAGAAATTACaTTTGATgTAAgtcctggtgTggTggc | AAAaagAaaAAGAAATTACcTTTGATaTAAtaatatactTatTtaa
6_71665     : AgAaAaAgAatagTATAaAaATATATcacgcCTGtaATcccAgcAC | AtAtAtAtAtataTATAtAtATATATgtacgCTGgtATaatActAC
```

Additional file 4. Sequences around 6-bp assumed TSDs. Nucleotides identical between 5' and 3' junctions are in uppercase. TSDs if we allow a 1-bp nucleotide substitution sandwiched by ≥2-bp stretches of homologous nucleotides are shaded in yellow.
